# Laser‐Patternable and Stretchable Metal Electrodes Using Metal–Amine Coordination Complexes

**DOI:** 10.1002/adma.202506722

**Published:** 2025-08-29

**Authors:** Seongyu Lee, Ngoc Thanh Ho, Jin Hong Kim, Gumin Kang, Hyungduk Ko

**Affiliations:** ^1^ Nanophotonics Research Center Korea Institute of Science and Technology Seoul 02792 Republic of Korea; ^2^ KHU‐KIST Department of Converging Science and Technology Kyung Hee University Seoul 02447 Republic of Korea

**Keywords:** coordination bonding, laser patterning, stretchable electrode, thin metal electrode

## Abstract

Coordination bonding is a crucial interaction between heteromaterials that enhances both mechanical toughness and stretchability, with mussels serving as a natural example of thriving in harsh marine environments due to this interaction. However, stretchable electronic materials based on this fundamental interaction have been rarely reported. In this study, a stretchable electrode, called the metal–amine coordination‐complex‐based electrode (MACE) is introduced, which involves the formation of coordination complexes between a solid metal and an organic layer. MACEs are based on a single Au layer with a thickness of a few tens of nanometers, yet they exhibit excellent stretchability of up to 70% and high durability under strain at 40% for 10 000 cycles without conventional treatments, such as pre‐stretching the substrate. Additionally, the direct laser patterning process on the metal film allows for high versatility in forming desirable patterns and adjusting stretchability. Furthermore, by utilizing the mechanical and electrical properties of MACE, a reversible soft actuator with a simple laminated structure is demonstrated. This approach, based on the formation of coordination complexes between heteromaterials, provides insights into fully mechanically stretchable electronics that achieve both softness and toughness simultaneously.

## Graphical abstract

A stretchable electrode, called the metal–amine coordination‐complex‐based electrode (MACE), is introduced, which involves the formation of coordination complexes between a metal and an organic layer. MACE exhibits excellent stretchability of up to 70% and stability under both air and wet conditions. Through direct laser processing, MACE forms desirable patterns under dry conditions and allows for the adjustment of stretchability.

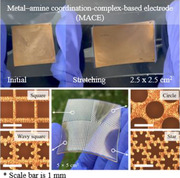

## 1 Introduction

Mussels are representative marine organisms that are known to contain metal ions that form coordination bonds with the core proteins of their byssus, which gives them the strength to attach to rocks and the ability to elongate without breaking when exposed to rough oceanic waves.^[^ ^1^, ^2^ ^]^ These abilities make mussels a valuable model for the development of materials with high tensile strength and toughness. In general, coordination bonding is a useful method for binding metals and organic groups by sharing electrons of organic atoms (O, N, S) or organic ligands with the *d*‐band orbital of a metal,^[^ ^1^, ^3^, ^4^, ^5^ ^]^ and this interaction has been intensely used to provide additional functionalities to nanomaterials or create chelate compounds.^[^ ^6^, ^7^ ^]^ In typical chemical reactions, bonds are formed at single reaction points; however, coordination interactions can involve the bond between metal and organic atoms (and/or ligands) at more than one, called coordination complexes.^[^ ^1^ ^]^ These complexes can also form the planarized coordination beyond a single bond. These features provide both high mechanical strength and high flexibility, as seen in mussels. The presence of coordination bonds in the structure, in addition to normal covalent bonds, enhances stability and mechanical strength to the overall chemical structure. Under excessive strain, the coordination bonds tend to break before the covalent bonds that form the main framework, thereby exhibiting high toughness.^[^ ^1^ ^]^ Thus, the tensile properties afforded by coordination bonds offer new possibilities for materials with high flexibility and stretchability; however, stretchable and conductive materials that ensure mechanical properties based on coordination complexes have rarely been reported.

In particular, stretchable electrodes that consist of the conducting materials and stretchable organic template have been intensively researched as an important component in the various fields of soft bio, optoelectronic devices, etc. by using many types of materials (such as metal, transition metal oxide, conductive organic materials, and multiple combinations thereof).^[^ ^8^, ^9^, ^10^, ^11^, ^12^, ^13^, ^14^, ^15^, ^16^, ^17^, ^18^ ^]^ However, compared to the successfully commercialized electrode, indium tin oxide, the insufficient conductance of next‐generation electrodes presents a significant problem. This issue makes it difficult to utilize stretchable electrodes in electronics. Indeed, many alternatives have been applied in similar applications that monitor the resistance change under strain, where high conductivity and low resistance are not necessarily required.^[^ ^19^, ^20^, ^21^ ^]^ Thus, there is still significant demand to ensure high conductivity for practical electronic device applications.

Among the stretchable electrode candidates, metal‐based electrodes, particularly metal film electrodes including mesh type, exhibit excellent conductivity and the practical possibility of fine patterning compared with other types of conductive materials.^[^ ^22^, ^23^, ^24^, ^25^ ^]^ However, the weak interaction between metal conductors and organic templates presents a significant problem in ensuring mechanical strain.^[^ ^15^, ^26^ ^]^

To improve the mechanical properties of electrodes based on metal, the approaches regarding the interfacial interaction between the metal and stretchable organic template have been studied by incorporating functional materials.^[^ ^15^, ^26^ ^]^ Although recent studies emphasize the significance of the interface between the main conductive metal and the elastic substrate and introduce the novel materials, the interfacial effects of supporting layers such as Ti or Cr—used prior to the deposition of the main conductive metal in vapor deposition methods—have rarely been addressed, hindering a clear understanding of the practical interface and minimizing the influence of these strong adhesive layers.^[^ ^15^, ^25^, ^26^ ^]^ Alternatively, the multi‐stacked electrodes with a couple of conductive materials have been adopted to form an electrical bypass to achieve the electrical and mechanical properties.^[^ ^21^, ^26^ ^]^ However, the multi‐stacked electrodes complicate interfacial interaction between the layers and their impact on stretchability.^[^ ^21^, ^26^ ^]^ Recently, Ga‐based liquid metals are emerging stretchable candidates;^[^ ^27^, ^28^ ^]^ however, the penetration of Ga into other metals and metal oxides, forming alloys, and altering physical properties,^[^ ^29^, ^30^, ^31^, ^32^ ^]^ must be avoided to ensure widespread applications. Therefore, a new perspective on the fabrication and materials of stretchable electrodes is still demanding.

In this study, we introduce a stretchable electrode, called the metal–amine coordination‐complex‐based electrode (MACE). This utilizes coordination complex bonding in‐plane with a thin Au layer of a few tens of nanometers, combined with an amine‐containing polymer. Coordination bonds in the metal‐organic layers are formed on the metal using solution‐processable organic materials, and then transferred to a polydimethylsiloxane (PDMS) elastomeric template through the exfoliation process. MACE exhibits low electrical properties, comparable to that of a fresh metal film, along with exceptional mechanical stretchability of up to 70%, while maintaining excellent durability in air and water. This strategy provides additional options for easily replacing the organic template and implementing laser‐induced patterning with mask‐free under dry conditions. By utilizing MACE's mechanical and electrical properties, we introduced the laminable grid electrode as a component that efficiently transfers carriers, and demonstrated novel applications of the combination with and shape memory electrodes. We believe that coordination bonding to heteromaterials provide a practical solution to the challenges associated with stretchable and soft electronics.

## 2 Results and Discussion

### 2.1 MACE Fabrication through Exfoliation Process

**Figure 1:**
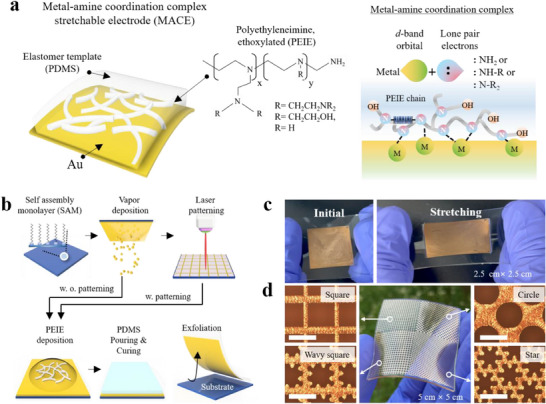
a) Schematic of the structure of MACE and the formation of a coordination complex with an amine‐containing polymer (PEIE) in the solid state. b) Overall procedure of MACE fabrication. c) Photographs of MACE, the size of which is 2.5 cm × 2.5 cm before and after stretching. d) Photographs of the laser‐patterned MACE processed at once, featuring four different patterns: square, circle, wavy square, and star shapes. The scale bar is 1 mm.

To form the coordination bonding between metal and organic materials the metal must have *d*‐orbitals to form the coordination bonds, i.e., it should be a transition metal. Among transition metal candidates like Ag, Au, and Cu, we chose Au because it is the most malleable and ductile.^[^ ^33^ ^]^ The organic anchor must contain elements that can graft onto the metal surface, possessing lone electron pairs and less electronegativity.^[^ ^1^ ^]^ Additionally, the polymer type was adopted because it can propagate the stress into the polymer chains and support the metal film. Polyethyleneimine (PEI) is a representative amine polymer, consisting of sufficient N, that is fully saturated and mechanically flexible, but exhibits low chemical stability.^[^ ^34^, ^35^ ^]^ To ensure chemical stability and reliability, we utilized a more oxidized form, ethoxylated polyethyleneimine (PEIE), in which the majority of the amine groups are converted to tertiary amines, as described previously (**Figure** **1**a).^[^ ^34^ ^]^ As a further step to minimize the effect of ions that disrupt the interfacial coordination bonding,^[^ ^1^ ^]^ we introduced the more oxidized formation of PEIE (Ox‐PEIE). In Ox‐PEIE, residual water molecules that protonate amine groups were eliminated from the polymer matrix, increasing the number of atoms available for bonding with the metal. In the next section, the mechanical properties of the MACE were assessed depending on the types of the PEIE.

Figure 1b shows the overall process of MACE fabrication based on exfoliation from the substrate. In the first step, the substrate was treated with a self‐assembled monolayer using dodecyltrichlorosilane (DTS) units to reduce the adhesion of Au to the substrate and facilitate effective exfoliation. Subsequently, Au with a thickness of less than 50 nm was deposited by vapor evaporation as shown in Figure S1 in Supporting Information, and PEIE layer was formed by solution‐process. As reported previously, the mechanical stretchability of stretchable electrodes is attributed to the properties of the organic substrate.^[^ ^20^, ^21^, ^23^, ^26^, ^36^, ^37^, ^38^ ^]^ Among them, PDMS was adopted as an elastomer template. It was formed by pouring on the metal/organic layer, followed by curing. Thereafter, the MACE was obtained by exfoliating stacked films from the substrate (Movie S1, Supporting Information). This fabrication procedure uses a reverse approach, stacking an elastomeric substrate on a conductive material layer. This fundamentally circumvents thermally induced oxidation of organic templates and deviation of metal growth depending on the substrate when the metal is directly deposited onto an organic template.

Optically, the transmittance of MACE, where Au film was transferred, was almost identical to that before exfoliating (Figure S2, Supporting Information). Moreover, this process can be extended to other metals such as Ag and Cu that have a d‐band, but not for Al (Movies S2–S4, Supporting Information). Figure 1c shows photographs of MACEs based on Au depending on stretching. This is a notable observation when compared to the case without the PEIE layer, where the bare Au film could not be uniformly transferred (Figure S3, Supporting Information). For a patterned MACE (p‐MACE), the patterns were drawn directly through laser processing before PEIE deposition, and subsequent processes were conducted in the same manner. Figure 1d shows optical microscopy (OM) images of p‐MACE fabricated through laser‐induced patterning, featuring a variety of shapes, including squares, circles, wavy squares, and stars. This is the first report that pure metal‐based stretchable electrodes are patterned using a mask‐free method under dry conditions.

### 2.2 Morphology of MACE and Surface Interaction between Au–Amine

**Figure 2:**
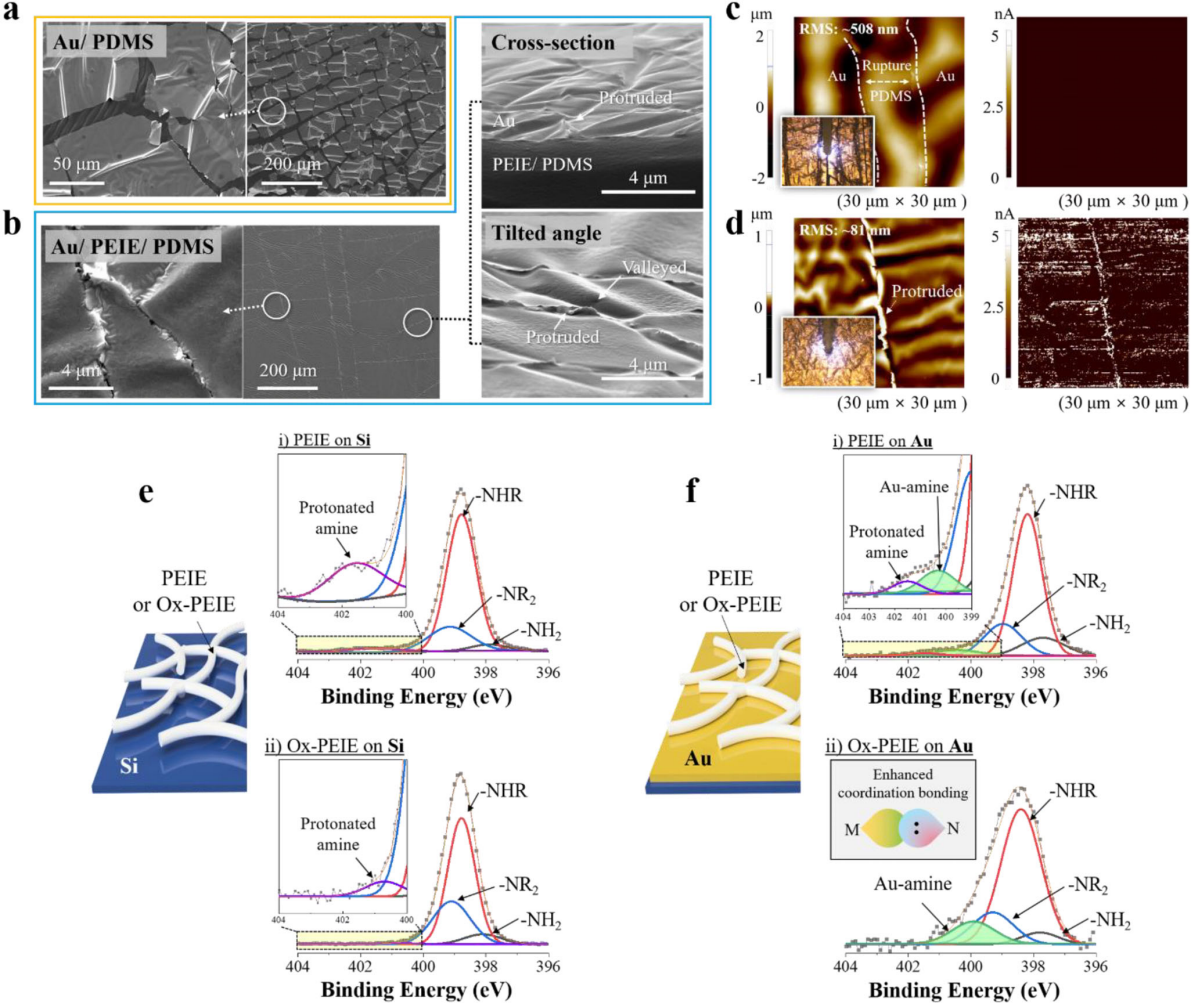
a,b) SEM images of the transferred Au surface with and without PEIE. The yellow box indicates the absence of PEIE, while the blue box indicates its presence. Cross‐sectional and tilted views were examined for the MACE samples. c,d) AFM images of the topography (left) and conductivity (right) of the transferred Au film without and with PEIE. The surface roughness was estimated to be ≈508 and 108 nm without and with the PEIE layer. The scanning size is 30 µm × 30 µm. e,f) N1s XPS analysis of the bare PEIE and Ox‐PEIE on Si wafer and Au film. The measured data are represented by the gray square. Each color line corresponds to a specific bond, with the orange line representing the cumulative line.

The coordination bonding supported by PEIE induced a significant difference in resulting films. Generally, the Au films transferred onto PDMS showed a buckled and wrinkled morphology (Figure S3, Supporting Information), which was attributed to the surface morphology of bare PDMS (Figure S4, Supporting Information).^[^ ^39^, ^40^, ^41^ ^]^ However, the Au film transferred on PDMS without PEIE showed severe cracks and voids, separating into Au flakes because the weak interaction between metal and PDMS (Figure S3a,b, Supporting Information).^[^ ^26^, ^40^, ^41^ ^]^ These severe damages were clearly observed in the scanning electron microscopy (SEM) images shown in **Figure** **2**a.

In contrast, in the case of PEIE, the entire Au film was successfully transferred to PDMS, providing experimental proof that PEIE forms strong adhesion with both Au and PDMS. Trace marks appeared in the middle of the MACE (Figure 2b and Figure S3c,d, Supporting Information), which were also observed on the bare PDMS and attributed to the local stretching of PDMS in a direction perpendicular to the exfoliation during the peeling‐off process (Figure S4, Supporting Information).^[^ ^41^ ^]^ Furthermore, as the concentration of PEIE increased, the transferred Au exhibited deeper wrinkling and buckling morphologies, resulting in fewer visible Au boundaries (Figure S5, Supporting Information). This indicates that the rough surfaces of PDMS and PEIE/PDMS were projected onto the thin Au film, demonstrating that the compressive force was sufficient to buckle the Au film during the exfoliation of MACE. The cross‐sectional image clearly shows that the Au was tightly bonded to the PDMS, with the boundary between the Au and PEIE/PDMS being scarcely visible as shown in Figure S1 (Supporting Information). The tilted view further confirms that the folded or protruded Au domains are physically connected, ensuring continuous electron transport channels (Figure 2b).

To confirm the conductance across this thin metal film, we performed conductive atomic force microscopy (AFM). The topology (left) and electrical conductance (right) were recorded on the same surface of each Au film (Figure 2c,d). In the absence of PEIE, a permanent rupture and a high roughness of ≈508 nm were observed, with no electrical properties detected on that surface (Figure 2c). In contrast, in the case with PEIE, the surface roughness was reduced to ≈108 nm, and electrical flow was observed (Figure 2d). These findings confirm that the presence of PEIE yielded unprecedented results, both morphologically and electrically. As a further step, we introduced Ox‐PEIE, which has a lower pH, by removing water molecules that protonate amines and generate hydroxyl groups as shown in Figures S6 and S7 in Supporting Information. This material allows for more efficient interaction with Au by minimizing ion interference near the amine groups. By using this, we demonstrated Ox‐PEIE‐based MACEs by replacing PEIE. When comparing the morphological differences of MACEs based on the types of PEIE, no meaningful changes in the surface morphology were observed; rather, the results appeared nearly identical to those obtained using bare PEIE.

To understand how this approach efficiently transfers the metal film, X‐ray photoelectron spectroscopy (XPS) was conducted. Notably, materials like PEIE, which form a dipole moment on the surface, can affect the binding energies of elements.^[^ ^42^, ^43^ ^]^ Therefore, to investigate the binding energy changes of PEIE due to interfacial interactions, samples were carefully prepared, and further details of the XPS analysis are discussed in Supporting Information. Figure 2e shows the N1s analysis of the bare PEIE on silicon. The primary, secondary, and tertiary amines (‐NH_2_, ‐NHR, and ‐NR_2_) were observed at 397.9, 398.8, and 399.2 eV, respectively.^[^ ^44^, ^45^ ^]^ Additionally, a peak attributed to the protonated amine was observed at 401.6 eV because amines of PEIE are intrinsically protonated by water molecules. For the Ox‐PEIE, the corresponding amine peaks appeared at 398.1, 398.8, and 399.1 eV; however, the amount of the protonated amine, whose peak appeared at 400.7 eV, was negligible because of the elimination of residual water molecules, as observed in the pH test shown in Figure S6 (Supporting Information). When both PEIE types were formed on the Au surface, the bare PEIE showed a new peak at 400.3 eV and a decrease in the amount of protonated amines, which was attributed to the coordination bonding between the metal and the amine groups observed in the region of 399–401 eV.^[^ ^46^ ^]^ Notably, as expected, the Ox‐PEIE on Au showed a significant enhancement of the metal–amine interaction at 399.9 eV compared with that in bare PEIE (Figure 2f). This observation implies that charged ions generated by residual water molecules screen the amines that participate in the coordination bonding; thus, removing disrupters is an effective and practical way to enhance coordination bonds with the Au surface, and the Ox‐PEIE forms a stronger interaction with the metal. Furthermore, this bonding at their interfaces affected the Au counterpart, shifting the binding energy of Au to lower energy (by approximately 1 eV) in Au 4f analysis as shown in Figure S8 (Supporting Information). This result is similar to the phenomenon when Au film is covered by the materials capable of forming the coordinating bonding by available atoms such as N and S.^[^ ^47^, ^48^ ^]^ Probably, it resulted from the coordination bonds formed by donating the lone‐pair electrons of amine to Au, inducing a dipole moment on their interfaces and reducing the work function of Au. This ultimately shifts the entire energy bonding spectrum to a lower level.

In conclusion, the generation of metal–amine bonding and the shifting of Au 4f peaks suggest the formation of strong interactions between PEIE and Au. This highly encouraging observation demonstrates that coordination bonds can indeed be formed in solid‐state interfaces via simple solution‐based deposition.

### 2.3 Electrical Properties and Stretchability of MACE

**Figure 3:**
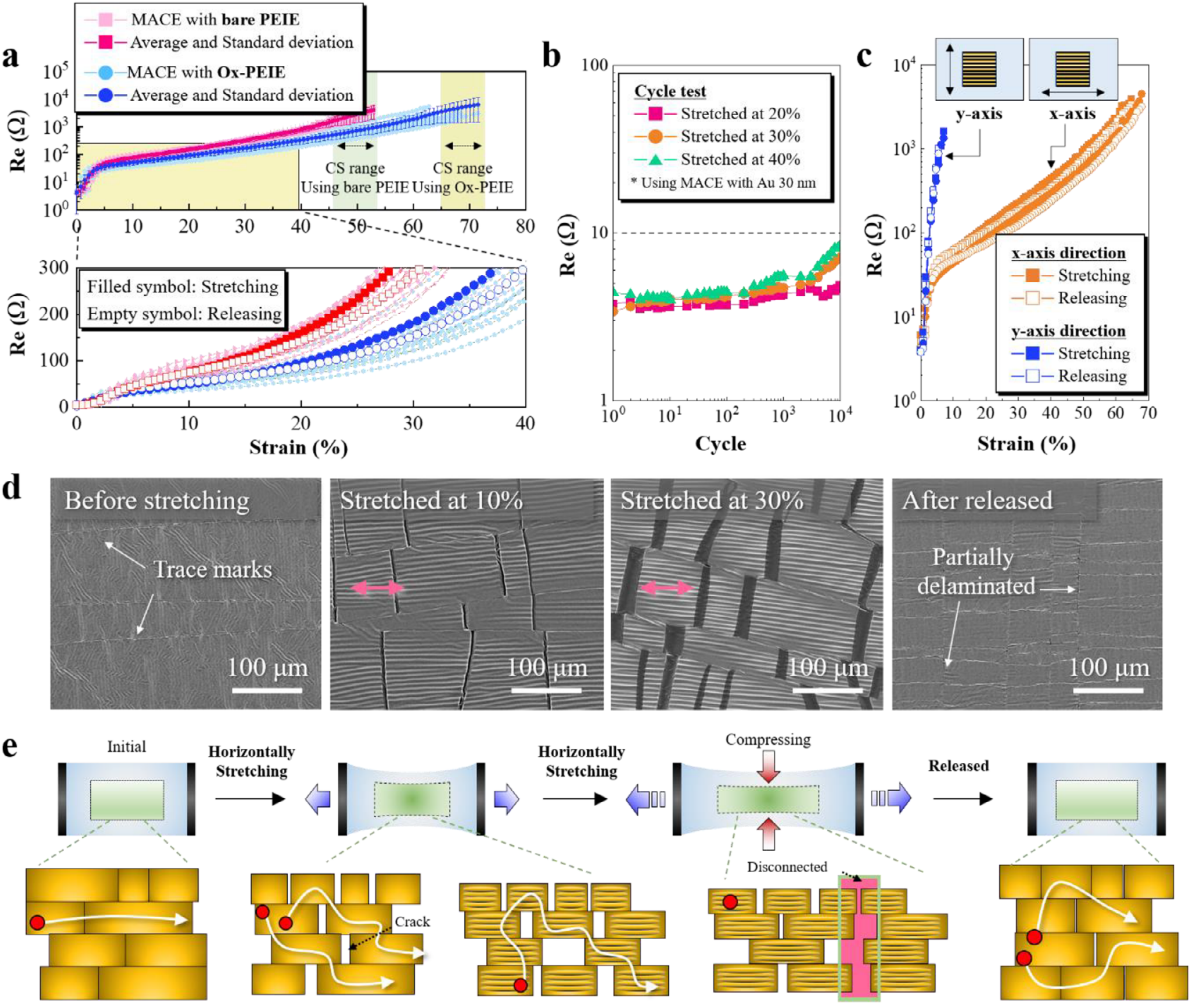
a) R_e_ and strain curves of MACEs fabricated using bare PEIE and Ox‐PEIE, and color symbols and vivid color symbols indicate results from more than 15 samples and their averages, respectively. The yellow region is enlarged below, and filled and empty symbols indicate each MACE when it was stretched or released, respectively. b) Durability test of MACE with a thickness of 30 nm. The MACE was stretched and released at 20%, 30%, and 40% strain. The cycle of stretching and releasing was approximately 1 Hz. c) Comparison of the uniaxial stretchability of MACE when it was stretched horizontally (x‐axis) or vertically (y‐axis) relative to the trace mark. The MACE was stretched horizontally or vertically in the direction in which it was exfoliated. Filled and empty symbols represent the stretching and releasing conditions. d) SEM images of unstretched MACE and MACE stretched at 10% and 30%, then released to the initial condition. e) Illustration of the MACE network when it was stretched and released. The magenta arrow indicates stretching direction.

**Figure** **3** shows the electrical and morphological properties of MACE against tensile strain. For precise electrical measurements, Ga/In eutectic electrodes (EGaIn) were applied to the edge of the MACE to avoid physical damages and contact issues (Figure S9, Supporting Information). The electrical resistance (*R*_e_) of the MACE showed a negligible change compared with the Au film before transfer, and demonstrated excellent current flow capability up to 300 mA cm^−2^, with linear behavior against applied bias (Figures S10 and S11, Supporting Information). Furthermore, the surface of transferred Au on MACE exhibited a similar work function to bare Au film (Figure S12, Supporting Information). These results demonstrate that the transferred Au film maintains the intrinsic surface properties of Au even with a single and thin conductive metal layer, forming uniform electron pathways.

For the stretchable test, MACE was evaluated by applying horizontal strain (i.e., along the x‐axis) until the electrode no longer responded to a bias, defined as the critical strain, and then releasing it back to its initial state. The stretchability of MACE was investigated by adjusting the thicknesses of PDMS, Au, and PEIE. MACE showed low sensitivity to changes in PDMS thickness (Figure S13, Supporting Information). However, it was significantly affected by the thickness of Au: MACE with 20 nm Au was more sensitive to strain, whereas the 30 nm case exhibited high resilience under strain and relatively low resistance. In contrast, the 40 nm Au case was less sensitive but also less stretchable (Figure S14, Supporting Information). Furthermore, the concentration of PEIE was positively correlated with the maximum stretchability of MACE (Figure S15, Supporting Information), gradually increasing from approximately 10% to about 50%. This mechanical stretchability appears to be related to the number of amine groups that participate in coordination bonding and the coverage of PEIE on the Au surface. Based on these findings, an Au thickness of 30 nm and specific conditions were selected to achieve optimal stretchability.

To investigate the correlation between the intensity of coordination bonding and stretchability, based on the optimized conditions, we demonstrated Ox‐PEIE‐MACE by replacing bare PEIE with Ox‐PEIE, which exhibited stronger coordination bonding with Au than bare PEIE as discussed in XPS results. We then compared the maximum stretchability of MACEs during stretching and releasing, the average resistance and standard deviation for each case are presented in Figure 3a, and further detailed information is summarized in Table S1 (Supporting Information). The MACEs with a bare PEIE layer were stretched by 45–53%. This is a notable achievement, given the fact that the sample without PEIE was not even exfoliated uniformly and that stretchability was attained without any mechanical pre‐treatment of the substrate, such as pre‐stretching. Furthermore, the MACE with Ox‐PEIE remarkably stretched to a maximum of approximately 70% strain (Figure S16, Supporting Information). When compared to the highest stretchability reported for other stretchable electrodes fabricated from thin metal films or metal meshes, even though our MACEs are based on a few tens of nanometers thick and are not assisted by technical assistance, our results are comparable to those (Figure S17 and Table S2, Supporting Information), and achieve the stretchability that exceeds the maximum strain of ≈30% induced by human skin.^[^ ^27^, ^49^ ^]^ Furthermore, in the durability assessment under strains of 20%, 30%, and 40%, MACEs withstood 10 000 cycles of stretching and releasing, and in each case, the resistance change remained minimal, below 10 Ω, demonstrating excellent mechanical durability (Figure 3b). Under further vertical stretching (i.e., along the y‐axis) of the MACE, the electrode demonstrated limited strain resistance, achieving a stretchability of approximately 10% (Figure 3c). This directional dependence on stretchability is generally observed in electrodes with specific orientation of the conductive materials.^[^ ^50^, ^51^ ^]^ In our study, this result seems attributed to the morphological characteristics of the MACE, inducing trace marks (Figure S18, Supporting Information). The differences associated with the stretching directions are discussed in detail later in the next section.

To understand the stretchability of MACE, SEM was used to examine its morphology. The bare PDMS exhibited folded and protruding features, forming a micro‐wavy structure under stretching (Figure S19, Supporting Information). When the MACE was stretched, these morphological changes were mirrored in the tightly bonded Au film. Additionally, several micro‐cracks, oriented perpendicular to the stretching direction, appeared partially and randomly in the Au layer. As the MACE elongated, the spacing between Au domains gradually increased. In contrast, all of the Au domains were connected vertically because of the compression force of PDMS with a high Poisson's ratio of 0.49 (Figure 3d).^[^ ^52^ ^]^ These morphologies affect *R*_e_; when stretching the MACE up to 50% strain, the horizontal *R*_e_ increased rapidly by a factor of 200 compared to the initial value, while the vertical *R*_e_ approximately doubled even (Figure S20, Supporting Information). This result indicates that the current bypassed in a direction perpendicular to the stretching direction, and the electrons travelling distance increased with the strain. This trend continued until the electrical percolation threshold disappeared, indicating disconnection, and the resistance recovered when the strain was released (Figure 3e). In conclusion, we confirmed that MACEs stretched up to approximately 70%, demonstrating exceptionally stable resistance and high durability against tensile strain without template modifications.

### 2.4 Laser‐Induced Patterning of MACE

**Figure 4:**
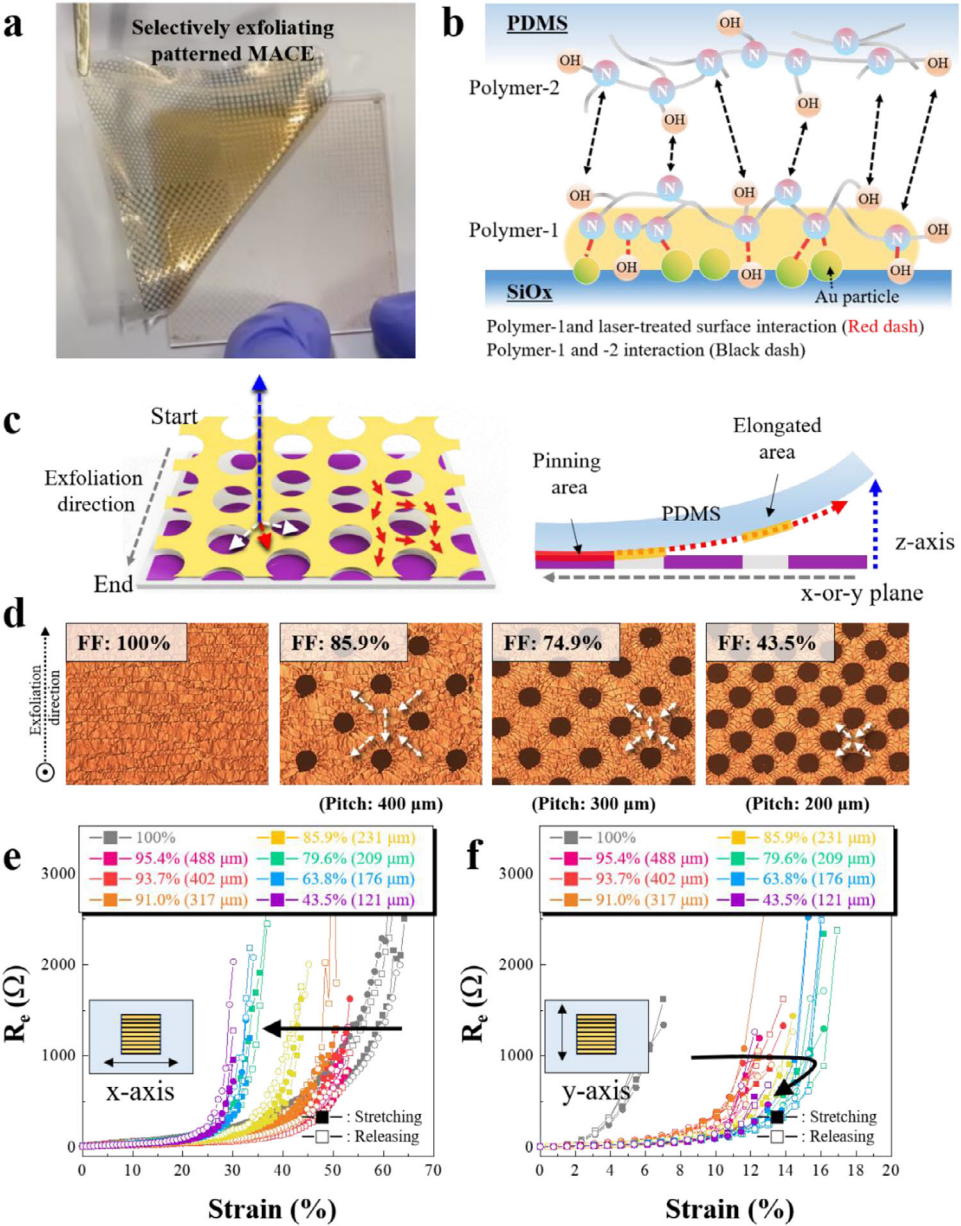
a) Photograph of the p‐ MACE with various patterns captured during the exfoliation process. b) Illustration of the selective exfoliation for the p‐MACE. Red and black dashes indicate the interaction between polymer‐1 and the laser‐treated surface and between polymer‐1 and polymer‐2, respectively. c) Illustration of the trace marks controlled through the arrangement of micropatterns. d) OM images of the MACE depending on FF of Au, which ranged from 100% to 43.5%. e,f) R_e_ and strain curves of the p‐MACE examined under x‐ and y‐axis stretching as a function of FF. The average edge‐to‐edge distance between hole patterns corresponding to each FF value is indicated in brackets.

To realize patterns, the crucial procedure is to distinguish the boundaries between the un‐patterned and patterned regions. In laser patterning process, many conventional studies utilize a change in the solubility of laser‐exposed areas, followed by a wet process under aqueous conditions.^[^ ^16^, ^17^, ^18^ ^]^ In the case of pure metals, although the laser process transforms the metal morphology from a film to particles, this physical change is not a fundamental factor that renders metal clusters soluble in chemical solvents. In contrast, in this study, the desired patterns were achieved by selectively exfoliating the unpatterned regions, and all processes were carried out under dry conditions. Considering these contrasting patterning mechanisms, this laser patterning technique represents the first report of a laser‐based method for patterning metal‐based stretchable electrodes under dry conditions (**Figure** **4**a and Movie S5, Supporting Information).

During laser processing, the metal film is not only aggregated into particles (Figure S21, Supporting Information) but also its surface becomes more hydrophilic due to thermally induced oxidation of the SAM‐treated surface (Figure S22, Supporting Information). Then, when PEIE solution cover both the patterned and un‐patterned areas, the polymer chains (polymer‐1) attach to the Au particles and Au film via coordination bonding; meanwhile, other polymer chains (polymer‐2) are anchored to the oxidized areas of the substrate (Figures S23 and S24, Supporting Information). These polymer chains interact with each other by the weak non‐covalent interactions like hydrogen bonding and Coulombic bonding, as well as entanglement between polymers. Thereafter, when exfoliating the stacked layers, polymer‐1 anchored on the SiO_x_ and polymer‐2 adhered to PDMS were separated, resulting in the selective transfer of the not laser‐patterned Au areas to PDMS. Meanwhile, Au particles covered by polymer‐2 were anchored on the substrate as residuals (Figure 4b). Transmittance spectra confirmed no significant implication by Au particles on the PDMS side after the exfoliation process (Figure S25, Supporting Information). Furthermore, SEM images showed distinguishable morphological changes of Au film depending on the laser patterning and exfoliation processes (Figures S26 and S27, Supporting Information). The patterning resolution can be significantly improved by using femtosecond pulsed lasers by minimizing patterning loss caused by the heat propagation (Figures S28 and S29, Supporting Information). We believe that the laser‐induced patterning technology offers the potential for stretchability, transparency, and device applicability.

Laser‐induced micropatterning provides interesting insight into the morphology control of MACE because the laser‐patterned areas have better interaction with a substrate, serving as localized pining sites. As discussed in Figure 3d, the MACE showed a significant difference in stretchability when stretched along the y‐axis than along the x‐axis. To minimize the directional stretch properties, we employed micropatterns as a manner to alter the stress direction of the Au film during exfoliation. By aligning the micropatterns, the trace marks are adjusted along the vector sum of the z‐axis (exfoliation direction) and the x‐ and y‐axis (fixed planar directions), as shown in Figure 4c.

For example, we created a simple micro‐hole pattern with a centered square array having a unit circle diameter of 140 µm. By adjusting the pitch from 400 to 200 µm between patterns, the various p‐MACEs were demonstrated, where the filling fraction (FF) of Au was estimated to be 85.9%, 74.9%, and 43.5% according to OM images (Figure 4d). When the FF was 100%, indicating no micro‐hole patterns, the trace marks formed linearly and predominantly along the x‐axis. However, when the FF was 85.9% with the introduction of micro‐hole patterns, the trace marks deviated from the linear pattern and exhibited local directional changes. This result shows that the introduction of micropatterns can alter the stress distribution during exfoliation. Furthermore, as the density of micro‐holes increased, the trace marks became more distinctly formed between the patterns. This result demonstrates that the introduction of micropatterns can control the stress direction during exfoliation, ultimately adjusting the electrical and mechanical properties of the film.

Figure 4e,f shows the strain‐*R*_e_ behavior of p‐MACEs with various FFs from 95.4% to 43.5% by adjusting the pitch and diameter of holes arranged in a c‐square array. For the p‐MACEs, the average edge‐to‐edge distance between hole patterns was estimated to range from 488 to 121 µm, and these values were indicated in each legend as a function of FF.

Each was evaluated in both the x‐ and y‐axis directions. The critical strain of the MACE under stretching along the x‐axis decreased from ≈65% to 53%, 45%, and 28% as the FF of Au decreased (Figure 4e). This degradation is inevitable, as the formation of patterned areas inherently reduces conductive regions and thus the percolation pathways. Interestingly, the critical strain in the y‐axis direction increased significantly with the formation of micropatterns (Figure 4f). In the case of FF of 100% with no patterns, the critical strain rose rapidly with increasing stress and it resisted ≈7% of strain in y‐axis stretchability. In contrast, the introduction of micropatterns greatly increased the critical strain. The MACE with the FF of 95.4% tolerated to ≈12% of the strain, and the critical strain improved to ≈17% even as the FF was reduced to ∼80%. However, with further reduction in FF, the stretchability decreased to ≈12% due to the reduction in conductive pathway. This result indicates that bidirectional stretchable conductive properties can be controlled by managing trace marks.

### 2.5 Long‐Term Stabilities of MACE in Air and Wet Conditions

**Figure 5:**
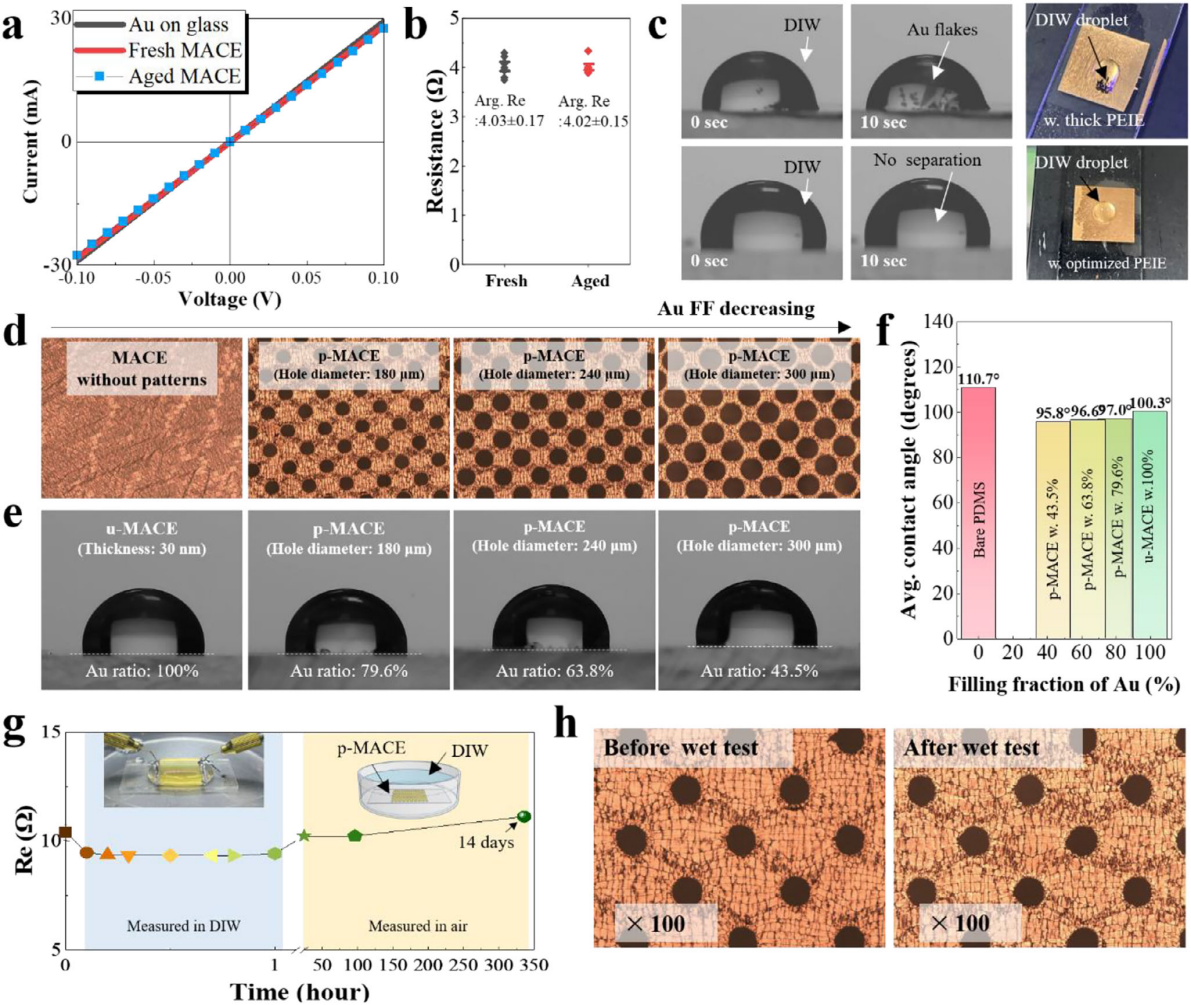
a) Comparison of the R_e_ of fresh and aged MACE. Aged MACEs were kept in ambient air conditions for six months. b) Averaged R_e_ of the fresh and aged MACE evaluated using more than ten samples. c) Photographs of MACEs with different thicknesses of PEIE over time when the single droplet of deionized water (DIW) was dropped onto MACE: the upper is for MACE with thick PEIE, and the lower is for MACE with optimized PEIE thickness. d,e) OM and contact angle images of un‐patterned MACE (u‐MACE) and patterned MACE (p‐MACE) as a function of the Au ratio. The Au area of MACE was adjusted from 100%, 79.6%, 63.8%, and 43.5% by varying the patterning area. f) Summary of contact angle results of p‐MACEs. g) Wet stability test of p‐MACE (2 cm × 2 cm) consisting of an Au area of 85.9%. h) OM images of p‐MACE before the wet test and after immersion in DIW for 14 d.

We evaluated morphological and electrical properties of fresh and aged MACEs that were stored for six months under ambient air conditions. OM images showed both fresh and aged MACEs appeared identical, with no meaningful signs of separation or exfoliation from the organic template (**Figure** **5**a,b). In the evaluation of R_e_ using more than ten samples, the fresh MACE exhibited a R_e_ of 4.03 ± 0.17 Ω, while the aged MACE showed a R_e_ of 4.02 ± 0.15 Ω, indicating no significant difference (Figure S30, Supporting Information). The long‐term storage stability of MACE is likely attributed to the nature of Au, which has a deep work function that inhibits oxidation.

In the evaluation of contact angle using the droplet of deionized water (DIW), PDMS and Au exhibited hydrophobicity, except for PEIE, which is a representative water‐soluble polymer (Figure S31, Supporting Information). These observations suggest that MACE could be destroyed by water due to the nature of PEIE. However, interestingly, the wet stability of MACE presented a significant difference depending on the thickness of PEIE (Figure 5c). For the MACE with thick PEIE, when the droplet of DIW fell onto the surface of MACE, Au film was rapidly exfoliated and destroyed into flakes, floating in water within a few seconds. It is likely that water permeated into the damage area of of Au film and dissolved the PEIE layer. In contrast, the optimized MACE with a thin PEIE layer withstood water exposure and maintained its contact angle. It is likely that when the surface properties of the thin layer become negligible, the wettability of the upper layer reflects the underlying bulk substrate in the stacked layers.^[^ ^53^, ^54^ ^]^ Indeed, the contact angle of Au varied depending on whether the substrate was PDMS or glass, even though the Au thickness was identical (Figure 5c and Figure S31, Supporting Information). For the p‐MACE, when the FF was reduced from 79.6% to 43.5%, the contact angle of the patterned samples slightly decreased from 100.3° to 95.8° due to the increased portion of the PEIE area. However, in all cases, relatively high hydrophobicity was maintained (over 90°), indicating that MACE can withstand wet conditions (Figure 5d–f).

The wet stability of MACE was evaluated by measuring its electrical resistance while submerging it in DIW and checking every ten minutes for up to one hour. Before submersion, the initial *R*_e_ was 10.4 Ω, which slightly decreased to 9.3 Ω in DIW, likely due to impurities, and remained stable throughout the 1 h evaluation. After drying, the R_e_ returned to its initial value. For extended observation, MACE was submerged for 24 h, maintaining a *R*_e_ of approximately 9.5 Ω. After 336 h (14 d), the *R*_e_ slightly increased to around 11.1 Ω (Figure 5g). Notably, MACE demonstrated significant stability even under prolonged submersion in water. After the long‐term wet test, no significant morphological changes were observed (Figure 5h). Thus, we concluded that MACE is highly resistant to water and can serve as a stable electrode.

### 2.6 Applications Using MACE

**Figure 6:**
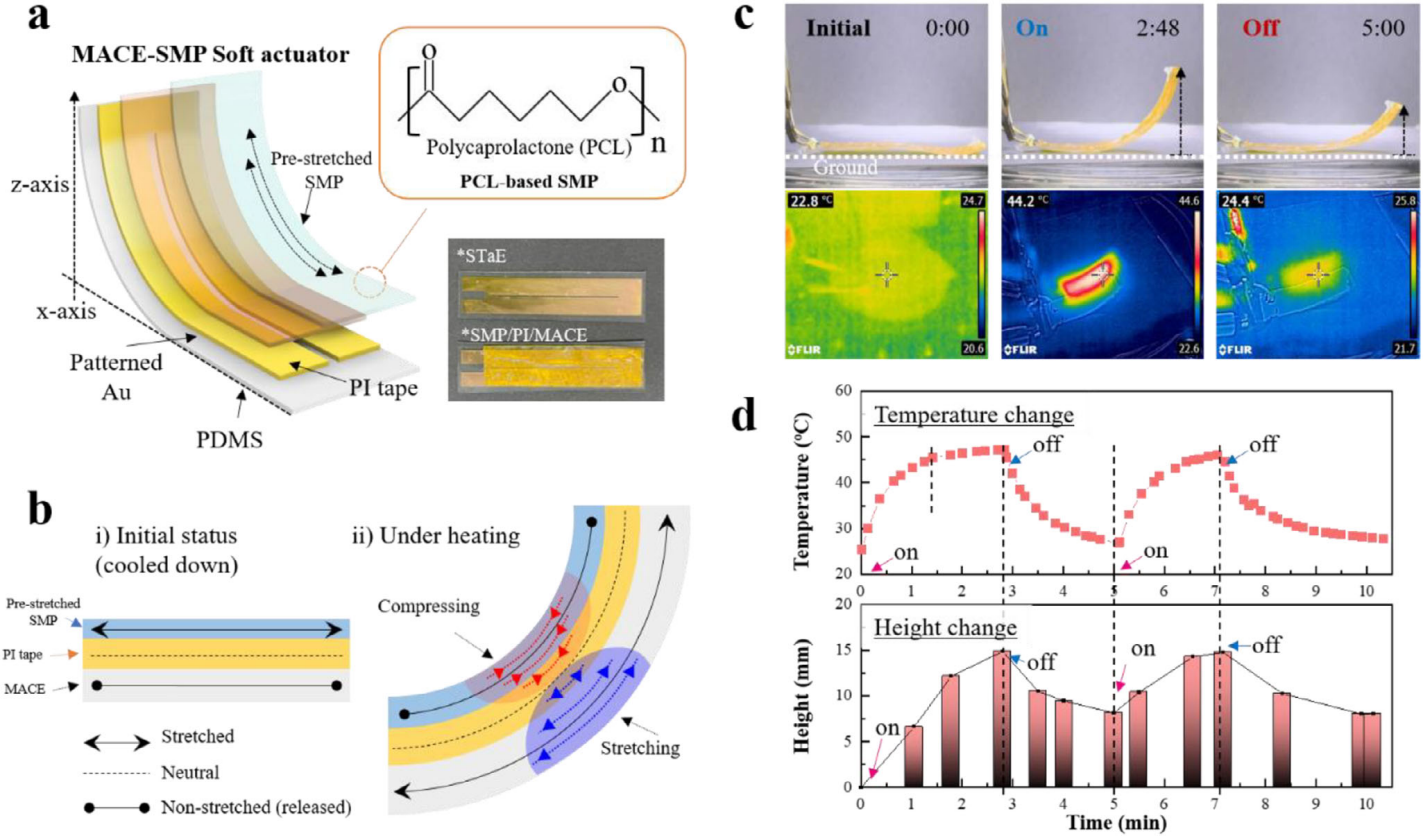
a) Scheme and photograph of a MACE‐SMP soft actuator, which was fabricated by laminating pre‐stretched SMP by about 200% and polyimide (PI) tape onto the patterned MACE. b) Illustration of the operating mechanism of the MACE‐SMP soft actuator under heating compared to its initial state. Under cooling, compressing and stretching were reversed. c) Photographs showing height changes of the MACE‐SMP actuator, along with corresponding thermal images for each condition (On and Off). In the On condition, a bias of approximately 2 V was applied, inducing a current flow of around 100 mA. In the Off condition, the actuator was cooled down to room temperature. d) Relationship between the reversible motion and thermal changes of the MACE‐SMP actuator over time, with each On and Off occasion indicated by arrows.

We leveraged the electrical and mechanical characteristics of MACE as innovative solutions to address existing challenges, rather than fabricating conventional applications that can be achieved using other stretchable electrodes. Based on MACE's tensile resilience and electrode characteristics, we demonstrated a thermally operating reversible soft actuator by combining MACE with shape memory polymer (SMP) (**Figure** **6**a). SMP stores external tensile stress in a glassy phase by being stretched and releasing it in a rubbery phase upon heating. Due to this characteristic, SMP is employed as a power source generating motion in soft actuators.^[^ ^55^, ^56^ ^]^ However, since SMP‐based actuators generate motion by consuming stored force, only a single operation is available unless additional force is applied for re‐stretching SMP.

In the MACE‐SMP soft actuator, upon heating (i.e., On condition), the SMP shrinks as the stored tensile stress in the pre‐stretched SMP releases, causing the actuator to be compressed and generate bending motion. Simultaneously, the compressive force produced by the shrinking of SMP stretches the PDMS of the MACE. Consequently, the stored tensile stress in the SMP is converted into stress that bends the PDMS. Once the heat is removed (i.e., Off condition), the SMP is re‐stretched due to the recovery of the PDMS, causing the MACE‐SMP actuator to move in the opposite direction until the restoring force of the SMP and the elastic force of the PDMS reaches equilibrium (Figure 6b). This reversibility is enabled by the mechanical stretchability of MACE, which allows the soft actuator to return from its compressed state to its stretched form—a capability that is not feasible with conventional flexible electrodes due to their lack of sufficient elasticity.

In our experiment, when the actuator was heated above 45 °C, it reached its highest point. Subsequently, when the bias was turned off (under cooling), it returned to the lower position. However, when cooled down, it settled in the middle of its motion range (Figure 6c and Movie S6, Supporting Information), indicating that the elastic force of the PDMS was insufficient to fully re‐stretch the SMP. Figure 6d shows the temperature and height of the MACE‐SMP actuator depending on the applied bias. The height and temperature changes exhibited identical behavior over time; however, returning to the initial state before heating proved to be challenging. This issue may be addressed through further material development and optimization of the physical properties of the SMP and MACE.

For an additional application, we utilized p‐MACE as a grid electrode in organic‐Si hybrid photovoltaics. The grid is an essential element for extracting carriers in electronic devices; however, it is challenging to implement due to the lack of suitable grid patterning technology that can be applied onto solution‐processable materials. We demonstrated the grid electrode by establishing coordination bonding between the pre‐formed p‐MACE and the underlying layer via lamination (Figures S32 and S33 and Table S3, Supporting Information). This coordination‐based lamination method, being attempted for the first time, allows damage‐free integration of the grid electrode. Through this approach, the power conversion efficiency was significantly improved compared to the case without the grid electrode. This approach can be further advanced by incorporating effective design strategies to maximize photocarrier generation and efficient collection, contributing to the simplified fabrication of energy sources. We believe that MACE, based on coordination bonding between metal and organic materials, can be utilized as an electrode in electronic devices and to support or sense the movement of humans or living organisms, and its versatility will expand in various fields.

## 3 Conclusion

By realizing the coordination bonding in a metal–amine complex in the solid state, we fabricated stretchable electrodes called MACEs. Furthermore, we reinforced the interaction between the heteromaterials of the MACEs by increasing the number of organic atoms participating in the bonding and eliminating the residual water molecules that inhibit surface interactions. As a result, the MACEs exhibit excellent conductance and resistance to tensile strain of up to 70%. We introduced direct‐laser patterning technology for MACE; this report is the first to demonstrate the patterning of stretchable electrodes without a mask under dry conditions. The stretchability of these electrodes can be further modulated by adjusting the propagation of stress under strain. Using MACE, we introduced a novel application combined it with shape memory polymer, demonstrating a reversible soft actuator. We believe that MACE, as a stretchable electrode, has the potential to contribute significantly to the fields of soft and bioelectronics. Our methods for forming coordination complexes between heteromaterials and demonstrating micropatterning using pulsed laser techniques offer valuable insights into the design and realization of novel materials.

## 4 Experimental Section

### Materials

Polyethylenimine, 80% ethoxylated solution (PEIE), gallium–indium eutectic (EGaIn), isopropanol (IPA, anhydrous, 99.5%), toluene (ACS reagent, ≥99.5%), poly[4,4′‐methylenebis(phenyl isocyanate)‐alt‐1,4‐butanediol/di(propylene glycol)/polycaprolactone], polycaprolactone diol (Mn 10000), and N,N‐dimethylformamide (DMF) were purchased from Sigma‐Aldrich, Inc., Co. Dodecyltrichlorosilane (DTS) was purchased from Tokyo Chemical Industry Co., Ltd. A polydimethylsiloxane (PDMS) Sylgard 184 kit was purchased from Dow Co., Ltd. Au (99.99%) was purchased from Taewon Scientific Co.

### Preparation of Solution, Substrate Treatment, and Elastotic Template for Stretchable Electrode

The surface of the substrate was treated using dodecyltrichlorosilane (DTS) to reduce the interaction with the substrate and the metal layer above. The glasses (or Si wafers) were cleaned by sonication in acetone and then in IPA for over 20 min. The DTS solution was diluted with toluene to a concentration of 5 vol%. For the DTS treatment, the prepared substrates were treated through UV‐ozone for 10 min for a more effective treatment. Thereafter, these glasses were immersed in the prepared DTS solution for 24 h. Furthermore, unreacted agents and residues after the reaction period were removed by rinsing in pure toluene solvent under sonication, and then the substrate was dried under N_2_ flow before use. The PEIE solution was prepared by diluting it with IPA at proposed concentrations and stirred for at least one day. For the elastotic substrate, the PDMS precursor was mixed with its cross‐linker agent at a ratio of 10:1 and kept in a low vacuum chamber to remove bubbles inside. When it is used, the PDMS mixture is poured and cured in a hot oven at 60 °C for 1.5 h. For oxidized PEIE (Ox‐PEIE), the bare PEIE (37% in water) was condensed by heating it on a hot plate at 200 °C for more than three hours with stirring. It was then prepared by diluting it in IPA.

### Fabrication of Metal–Amine Coordination Complexes‐Based Electrodes (MACE)

Au was deposited on the surface‐treated substrate using thermal evaporation under a high vacuum, at a pressure of at least 5 × 10 ^−7^ torr. The diluted PEIE solution was then deposited on top of the metal film by spin‐coating at 3000 rpm for 40 s. Next, it was annealed on a hot plate at 80 °C for 10 min to remove residual water molecules and to form strong coordination bonding complexes. Subsequently, the sample consisting of PEIE/Au/surface‐treated substrate was moved into a plastic petri dish, and a PDMS mixture was poured onto it and cured. Finally, MACE was peeled off from the substrate. In the case of patterned MACE, laser patterning was conducted after the metal deposition, and then the remaining procedures were performed in the same manner.

### Patterning Process by Pulsed Lasers and Patterned MACE

A nanosecond fiber laser was employed based on a master oscillator power amplifier, operating galvanic mirrors from Hauser Machinery Equipment Co., Ltd. Its wavelength of laser light is 1064 nm. Specific parameters were adjusted: frequency was over 20 kHz, patterning speed was around 50–200 mm s^−1^, pulse width was 0.1–10 ns, and the processing power of the laser was adjusted from 20–26 mJ mm^−2^ depending on Au thickness. The laser patterning process was directly conducted on a thin metal surface before the PEIE deposition. Then, the remaining steps were performed as usual. For the microhole‐patterned MACE with a centered square arrangement, we fixed a diameter of the unit circle at 140 µm and adjusted the distance of the pitch from 400 to 200 µm. For femtosecond laser, we utilized UFL‐300 model, L2K Co., Ltd. The output power of the fs laser was adjusted to below 0.01 W, with a center wavelength of 1030. The laser spot size was approximately 2 µm. The laser beam operated in a TEM_00_ mode with an M^2^ value less than 1.2. The repetition rate was 100 kHz, the pulse duration was 230 fs, and processing speed was 20 mm s^−1^.

### Electrical Resistance Measurement

For the electrical resistance evaluation, the exact size of the metal area, which was 1 cm×1 cm, was formed by patterning the deposited metal film with a nanosecond laser. The current–voltage (*I*–*V*) characteristics of the electrodes were evaluated using a probe station (Keithley, K4200). The electrical resistance was obtained using the equation of Re  = *V*/*I* , where Re represents electrical resistance, *I* is the current, and *V* is the applied bias. Prior to the electrical measurement, EGaIn was loaded at the edges. If the measuring tip directly contacts MACE, the contact resistance shows a significant deviation in Re, and it is frequently physically damaged due to the hardness of the tips because MACE is based on thin metal and soft materials. Small damage easily propagates inside and can lead to the permanent rupture of the template during stretching.

### Optical, Surface Characteristic, and Binding Energy Analysis

The transmittance of electrodes was measured using a Shimadzu UV‐3600 plus spectroscope with an MPC‐603A integrating sphere (Shimadzu Scientific Corp). A scanning electron microscope (Inspect F) was employed to characterize the surface morphologies of the metal film at an acceleration voltage of 10 kV. The X‐ray photoelectron spectroscopy (Thermal Fisher Scienctific, Nexsa) was conducted using a microfocus monochromatic Al Kα X‐ray source (1486.6 eV). Atomic force microscopy images inducing conductive images were obtained by XE‐100 (Park systems), and the scanning area is 30 µm × 30 µm.

### Stretchability Test

The Au film was deposited on the surface‐treated substrate, which had a size of 5 cm × 5 cm. Before the PEIE formation, the size of each Au film was confined to 1 cm × 1 cm by a nanosecond laser to ensure an exact measuring size and subsequent processes were conducted. To avoid the influence by exfoliation directions when peeling off MACE, the exfoliation direction constant was kept from bottom to top side unless otherwise specified. By exfoliating this way, the trace marks of MACE were reminded horizontally along to x‐axis. For the y‐axis stretchability test, MACE was fabricated by exfoliating it from left to right to form trace marks in the opposite direction. The MACE (after exfoliation) was then cut to 1.5 cm × 5 cm. Both sides of the template, around 1.5 cm × 1 cm, were used as a holding area using a jig. The stretchability of MACE was stretched to horizontally, which is parallel to trace marks (x‐axis) by increasing the strain stress up to defined as the critical strain, disconnected, and then releasing it to the initial point again. This cycle measurement accurately determines the stretchability and reliability by avoiding overestimation caused by the MACE escaping from its holder. MACE was stretched in the parallel direction (y‐axis) to the trace lines at a rate of 0.125 mm s^−1^. For the cycle test, the MACE was stretched at 20%, 30%, and 40%, respectively, and then released back to the initial state. The rate of the cycle was approximately 1 Hz.

### Soft Actuator Based on Shape Memory Polymer

For the shape memory polymer (SMP) film, 0.5 g of poly[4,4′‐methylenebis(phenyl isocyanate)‐alt‐1,4‐butanediol/di(propylene glycol)/polycaprolactone] and 0.2 g of polycaprolactone diol were dissolved in 5 mL DMF and stirred for 12 h. The mixture has a high viscosity. To ensure uniformity of the SMP dose and thickness, the prepared solution was poured onto a slide glass and spin‐coated at 300 rpm for 5 s. It was then transferred to a hot plate set at 90 °C and annealed for more than 30 min. After complete drying, it was peeled off from the substrate. The SMP film was pre‐stretched to 200% and fixed in the elongated state before attaching it to MACE. The MACE was patterned by laser to dimensions of 1.0 cm × 3.5 cm with an aspect ratio of over 3. The pre‐stretched SMP was fixed onto PI tape using common epoxy resin. Then, the SMP/PI tape was attached to the patterned MACE. To operate an SMP‐based soft actuator, heat was generated by Joule heating, gradually applying a bias of approximately 2 V, thereby flowing a current of about 100 mA. When the temperature reached over 40 °C, the actuator efficiently operated.

## Conflict of Interest

The authors declare no conflict of interest.

## Author Contributions

S.L.: conceptualization, data curation, formal analysis, investigation, methodology, visualization, writing—original draft, review & editing. N.T.H.: data curation, formal analysis, and investigation. J.H.K.: validation, writing–review & editing., G.K.: validation, writing–review & editing. H.K.: project administration, funding acquisition, supervision, writing – review & editing.

## Supporting information



Supporting Information

Supplemental Movie 1

Supplemental Movie 2

Supplemental Movie 3

Supplemental Movie 4

Supplemental Movie 5

Supplemental Movie 6

## Data Availability

The data that support the findings of this study are available from the corresponding author upon reasonable request.
