# Supplementary material for: Laser‐Patternable and Stretchable Metal Electrodes Using Metal–Amine Coordination Complexes
Source: Adv Mater. 2025 Aug 29;37(47):e06722. doi: 10.1002/adma.202506722 (PMC12651124; doi:10.1002/adma.202506722)
Supplement: Supplementary file 1 — Supporting Information [file ADMA-37-e06722-s005.docx]

**Supporting** **Information**

**Laser-patternable and stretchable metal electrodes using metal–amine coordination complexes**

Seongyu Lee^1^, Ngoc Thanh Ho^1^, Jin Hong Kim^1^*, Gumin Kang^1^*, Hyungduk Ko^1,2^*

**Contents**

**Figure S1-33**

**Table S1-3**

**Supporting** **notes**

UV-vis spectroscopy change depending on fabrication processes

Exfoliation depending on the presence of PEIE

Fabrication of oxidized PEIE

XPS analysis

XPS analysis of Au depending the presence of PEIE

Comparison in electrical and stretchability with previous metal-based stretchable electrodes

Reversible soft actuator based on MACE

Surface oxidation by laser illumination and enhancement adhesion with PEIE

XPS analysis of Au particles depending on the presence of PEIE

Laser-induced direct metal patterning

Transmittance spectra of Au particle film depending exfoliation processes

Morphology observation of Au film after laser patterning and before and after exfoliation

Estimation of filling fraction (FF) of microhole array

Lamination technology of patterned MACE in photovoltaics

**Movie S1-6**

Movie S1. Exfoliation process depending on the presence of PEIE

Movie S2. MACE with Ag

Movie S3. MACE with Cu

Movie S4. Exfoliation test using Al

Movie S5. Exfoliation of the patterned MACE

Movie S6. SMP actuator on and off (4x faster)

**
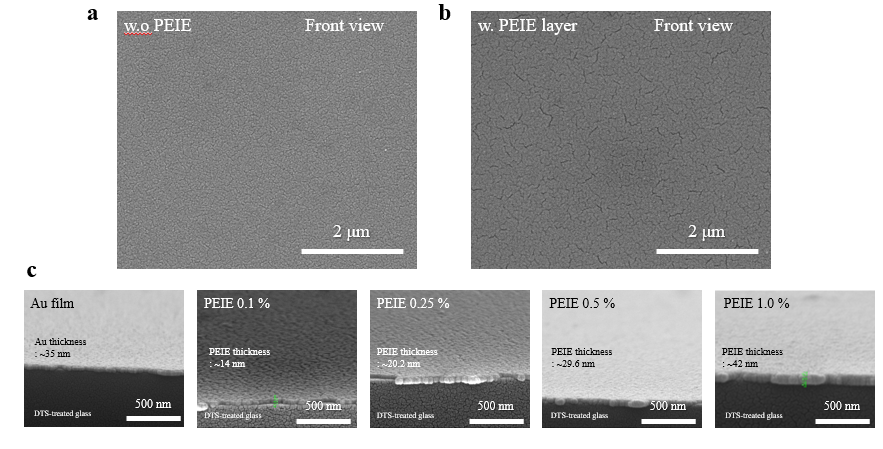
**

**Figure** **S1**. SEM images of a. Au film and b. PEIE layer on Au film. c. Cross-sectional view of the Au film covered by PEIE, with its concentration adjusted from 0.1 to 1.0 wt%. The PEIE thickness was estimated to be approximately 10 to 40 nm, determined by subtracting the Au thickness of 35 nm from the total measured thickness. The surface morphology of the Au film appears uniformly distributed across the entire surface, showing no significant changes even after PEIE deposition. In the cross-sectional view, the measured thickness increases gradually with higher PEIE concentrations.

**UV-vis spectroscopy changes depending on fabrication processes**

UV-vis spectroscopy reveals variations in the transmittance of Au and the substrates (glass and PDMS) depending on the fabrication process (Figure S2). The spectrum shows Au deposited with a 30 nm thickness on a DTS-treated glass substrate (green), which improves after coating with PEIE (purple). This enhancement is likely due to light interference caused by the higher refractive index of PEIE compared to air. After curing PDMS on the PEIE/Au and exfoliating the MACEs, the area previously occupied by the Au film becomes empty, indicating complete transfer to the PDMS side, which exhibits transparency almost identical to bare glass (cyan). In contrast, the Au film transferred to the PDMS side shows a spectrum similar to Au/PEIE (yellow), with slight differences near the 300 nm wavelength due to the substrate change from glass to PDMS. These transmittance spectra confirm the complete transfer of the Au film from glass to PDMS.

**
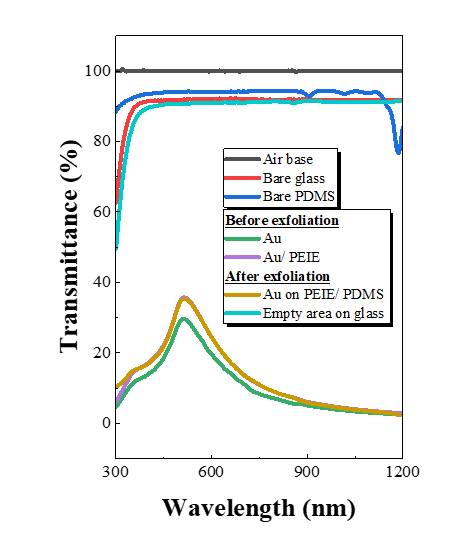
**

**Figure S2**. Transmittance spectra of the Au film following the processes of PEIE deposition and exfoliation.

**Exfoliation depending on the presence of PEIE**

In OM images of the Au and Au/PEIE films after PDMS curing, the surface morphology of the Au film observed through the PDMS appeared flat in both cases before exfoliation. Numerous dots were visible on the Au/PDMS film (Figure S3), likely due to adhesion issues between the metal and PDMS. In contrast, the Au/PEIE/PDMS film exhibited a clean, flat surface. After separating the PDMS from the substrate, a buckled and wrinkled structure formed in both cases. However, the presence or absence of PEIE led to distinct differences in the transfer of the Au film. The Au film directly transferred onto PDMS appeared fragmented, showing voids, damage, and boundaries between Au flakes (Figure S3a-b). In contrast, the Au/PEIE/PDMS film was fully transferred and exhibited unique trace marks, which depended on the direction of exfoliation (Figure S3c-d). These morphological changes resulting from the incorporation of an organic layer are very promising.


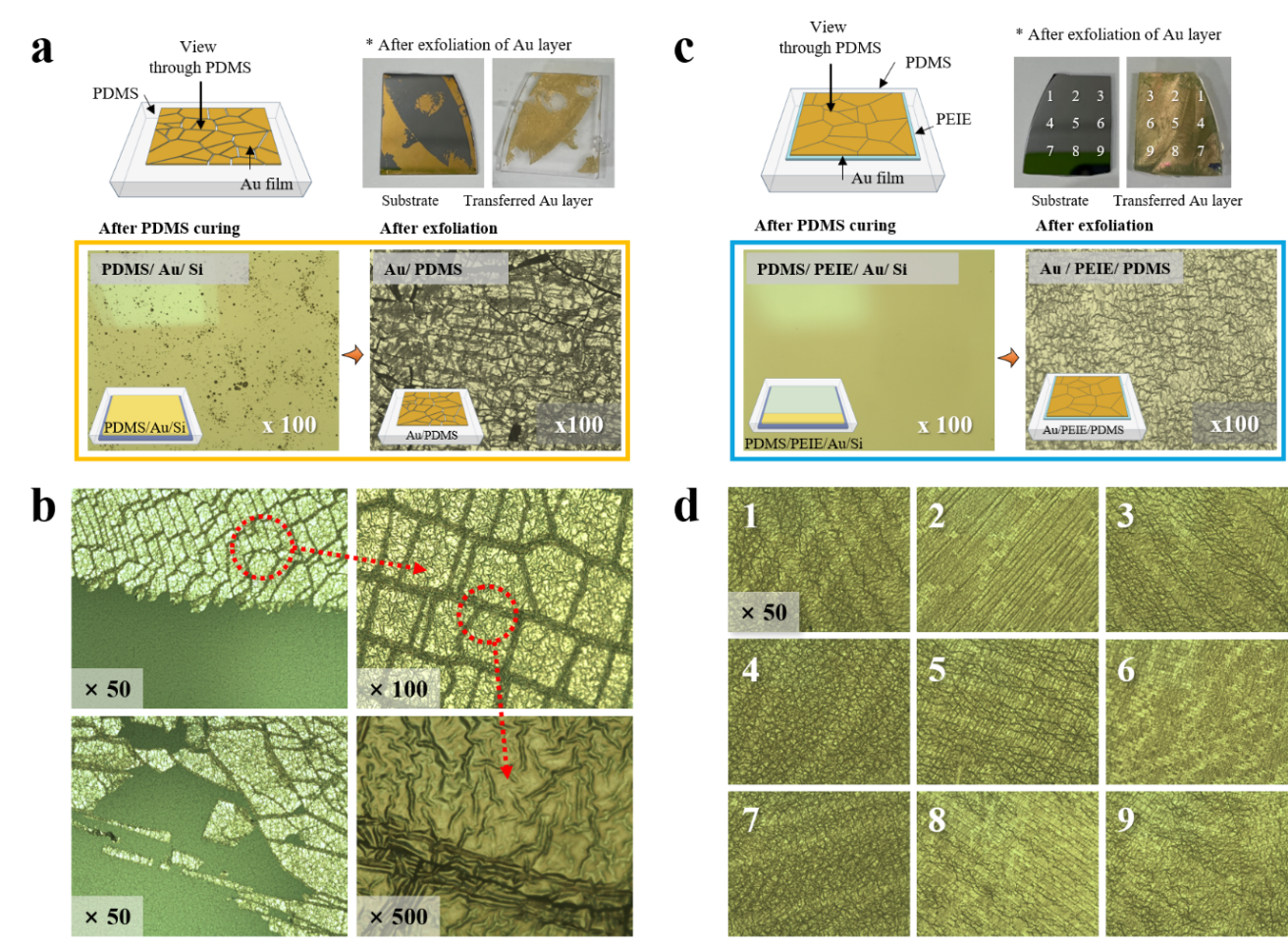


**Figure S3**. Schemes, photographs, and OM images of the transferred Au film on PDMS, depending on the absence or presence of a PEIE layer. a-b. Without PEIE. c-d. With PEIE. During PDMS exfoliation, the MACE process occurred randomly, without regard to direction.

These morphological changes, such as buckling and trace marks, appear to result from mechanical forces occurring after PDMS curing. These marks were also observed on the bottom side of the bare PDMS in a regular, perpendicular pattern, aligned with the exfoliation direction. However, they were not present on the top side (Figure S4). It seems these changes are caused by the local elongation of PDMS due to its interaction with the bottom layer and its elastic properties. These morphological changes are easily reflected on the thin Au film, which is only a few tens of nanometers thick. In this work, a self-assembled monolayer (SAM) was used to both cases to reduce surface interaction. However, the Au film still weakly interacts with the substrate due to imperfections in the SAM treatment and Van der Waals forces. When the PDMS precursor is poured onto the Au or Au/PEIE film and cured, the coiled chains of the PDMS precursor bond randomly with adjacent curing agents due to the flexibility of the saturated polymer. As PDMS cures, an interfacial interaction is established between the PDMS and Au film (or PEIE), counteracting the Au-substrate interaction. Once fully solidified, the compensating force accumulates within the cured PDMS chain, acting like a spring. Subsequently, when PDMS is separated from the substrate, the compensating force, which maintained the equilibrium between the Au-substrate and Au-PDMS interactions, is released. This causes the polymer chains in the PDMS matrix near the interface to be pulled inward, leading to the folding and protrusion of the transferred Au film. These processes seem to generate the trace marks observed after the exfoliation.


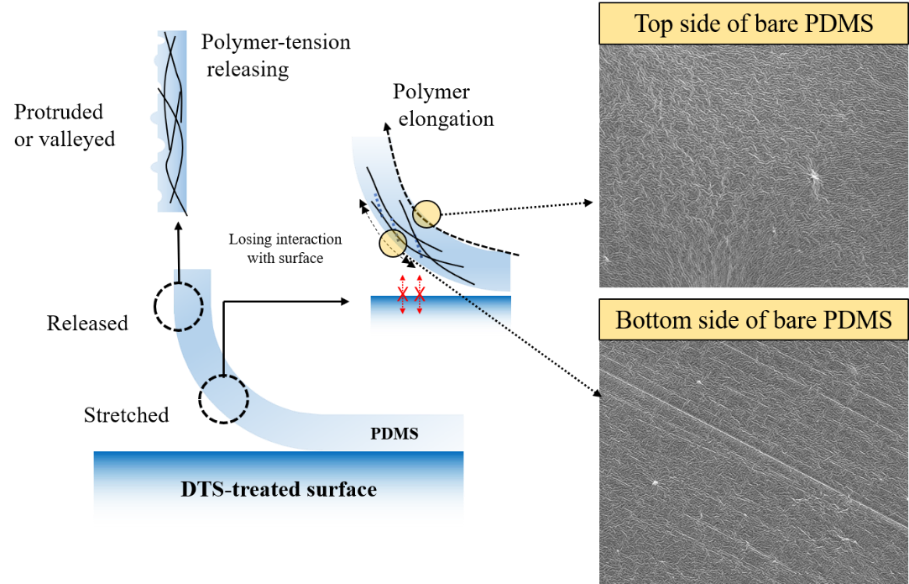


**Figure S4**. Exfoliation process of Au assisted by PEIE/PDMS from DTS-treated substrate, and SEM images of the top and bottom morphology of a bare PDMS after exfoliation.


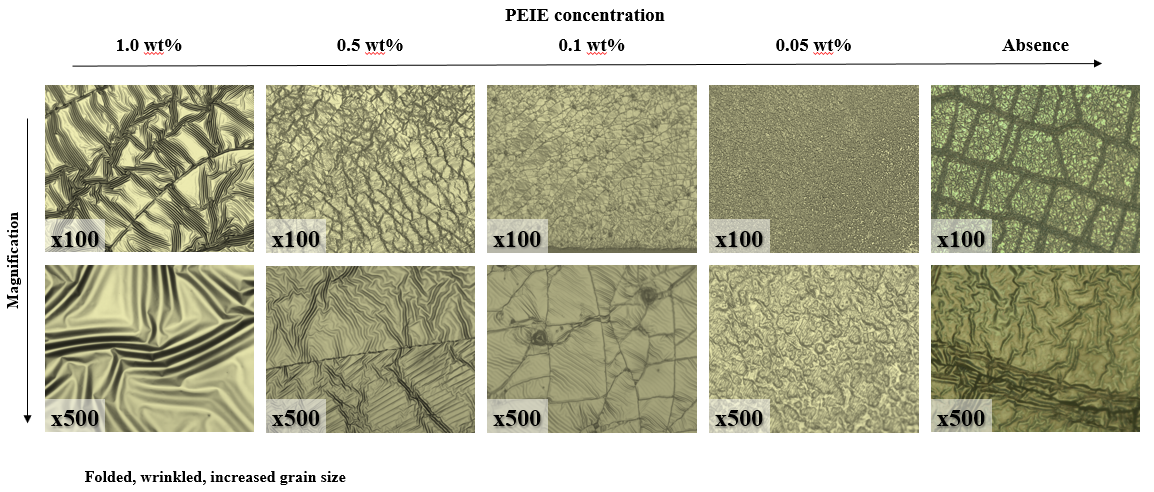


**Figure S5**. Optical images of the transferred Au film on PDMS depending on the concentration of PEIE.

**Fabrication of oxidized PEIE**

PEIE is a representative hydrophilic material that is naturally protonated by water, resulting in a basic solution.^[1]^ In defining the oxidation and reduction of PEIE, we considered bare PEIE, which is already protonated, as the reference state. When the residual water is eliminated from the polymer matrix, the number of hydrogen sources decreases due to the loss of water molecules. The condensed PEIE, after water removal, is defined as oxidized PEIE (Ox-PEIE) because it loses hydrogen.

To eliminate the residual water, 6 mL of bare PEIE polymer was prepared in a vial and heated on a hot plate set at 200 ^o^C for more than 3 hours. As time went by under heating, the total amount of PEIE was reduced by removing the water, causing the transparent solution to turn yellow and then brown. It is worth noting that previous works mentioned that a small dose of catalysis in the PEIE material is still contained in the stock solution at the PPM level.^[2]^ Therefore, it appears that an additional reaction occurred during the heating process, resulting in chemical structural changes that led to a color change in the PEIE solutions as residual water molecules were eliminated. To compare the two types of PEIE and Ox-PEIE, each was diluted into DIW at the same concentration of 1.0 wt% and the pH of the solution was measured using pH-indicator strips, which can measure pH in the range of 0-14. The bare PEIE exhibited a pH of 12-13, as seen in previous works,^[1]^ while the Ox-PEIE showed a pH value at least three orders of magnitude lower, ranging from 9-10 (Fig S6).

To investigate the chemical bond changes of bare PEIE Ox-PEIE, XPS analysis was conducted depending on the element (Figure S7). Each polymer was coated on a Si substrate. In C1s analysis, the C-C (peak 1) at 284.8 eV and C-N/C-O (peak 2) at 286.7 eV were defined in both cases as shown in Figure S7. The ratio of peak 2 over peak 1 improved from 8.8% to 16.20% in the case with Ox-PEIE compared to the bare case. N1s analysis showed the general amine peaks of primary (-NH_2_), secondary (-NHR), and tertiary (-NR_2_) amine consisting of PEIE. These peaks were defined at 397.9, 398.8, and 399.2 eV, respectively. There peaks were identical regardless of the type of PEIE. The broad protonated peak at 401.6 eV was detected due to the good familiarity with water. In Ox-PEIE, the protonation of the protonated amine was reduced, making it rarely recognized. However, there were no significant changes caused by additional chemical bonds in carbon, nitrogen, and oxygen analyses. Thus, it is concluded that the PEIE polymer chains were oxidized and condensed through the annealing process.


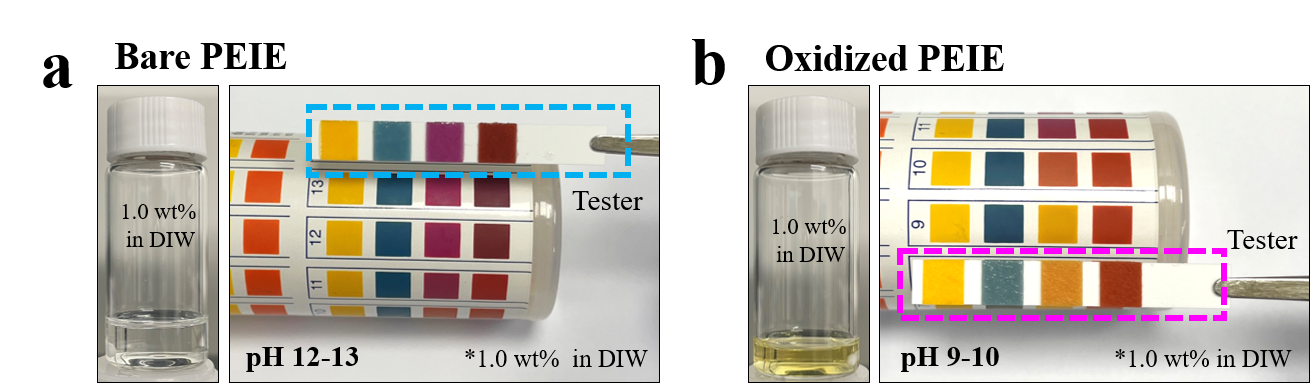


**Figure S6**. Photographs of PEIE solutions fabricated from a. bare PEIE and b. oxidized PEIE. Each polymer was dissolved in DIW at a concentration of 1.0 wt%. pH indicator strips were dipped into the respective PEIE mixtures. The bare PEIE exhibited a pH of 12-13, while the oxidized PEIE showed a pH range of 9-10.

**
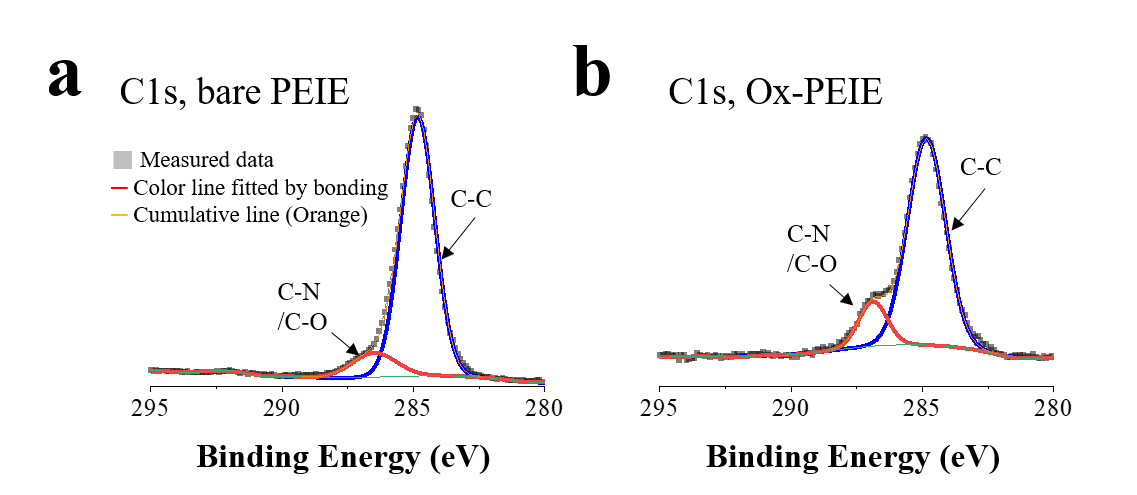
**

**Figure S7**. C1s XPS analysis of the bare and oxidized PEIE surfaces: a. bare PEIE and b. oxidized PEIE. Each PEIE sample was deposited on Si.

**XPS analysis**

In XPS analysis, the ultrashort wavelength of the rays is sufficient to extract the electrons from the inner shell, and the kinetic energy of the extracted electron is affected by the work function (WF) of the substrate.^[3,4]^ Usually, the binding spectra of the electrons from the measured sample were aligned by the adventitious carbon of C-C at 284.6-285.0 eV.^[3]^ However, when materials like PEIE, which form a dipole moment are deposited at the interface with a substrate, they can shift the vacuum level of the conductor (or semiconductor) thereby shifting the WF the alignment as well as accumulating extracted electrons.^[3,4]^ The Schottky barrier formed at the interface of conductive materials also affects the kinetic energy of the binding spectrum. These effects intricately influence the measurement of binding energy, making it challenging to align binding spectra using a singular criterion such as adventitious carbon or the Si peak. Furthermore, the properties of the substrate, such as its WF and type (metal or semiconductor), can also induce significant changes.

For these reasons, the comparison of the positions of measured peaks without considering the effects of the substrate and dipole layer can mislead the interpretation of bonds at the interface. Thus, for this XPS analysis, the criterion for alignment was adopted differently depending on the substrate: the adventitious carbon for the Si substrate and the pure Au peak for the Au film, respectively. When peak shifting occurs due to the deposition of PEIE, in these cases, the binding spectrum was additionally adjusted using well-defined chemical bonds based on previous literature. For N analysis, we utilized the peak of NH_2_ because it is the largest component of the PEIE matrix. For a better interpretation, we investigated the changes in the fraction of peaks and the generation of new bond by the presence of PEIEs by fixing the positions of assigned peaks.

**XPS analysis of Au depending the presence of PEIE**

To compare the relative interaction of Au and PEIE, PEIE solution was deposited on top of the Au film. The binding positions of sub-peaks near the main peaks were determined by their distances from well-defined peaks for better comparisons (Figure S8). For a fresh Au film, bulk Au 4f_5/2_ and 4f­ were detected at 84.0 and 87.8 eV,^[5,6]^ and surface-dominant peaks were observed shifted by 0.4 eV at 83.7 and 87.4 eV,^[6,7]^ and oxide peak peaks at 84.8 and 88.6 eV.^[8,9]^ When PEIE was introduced on Au, the overall peaks were shifted to lower energy by approximately 1 eV, while the fraction of sub-peaks seemed identical. This trend is a similar to findings reported in previous work when the Au film was grafted by amine or sulfur atoms, which form the coordination bonds with Au.^[5,10]^


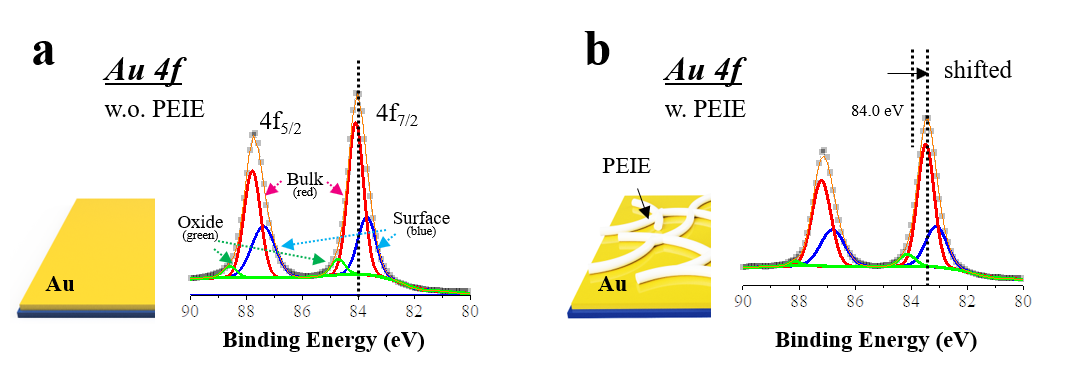


**Figure S8**. Au 4f XPS analysis of the Au film depending on the presence of PEIE: a. bare Au film and b. PEIE-covered Au film. The gray squares represent the measured data, while each colored line corresponds to a specific bonding: red for bulk, blue for surface, and green for oxide formation of Au. The orange line indicates the cumulative spectrum.


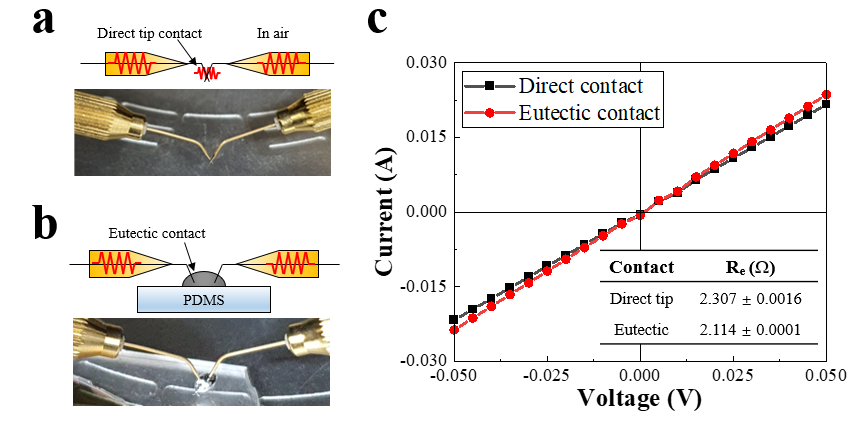


**Figure S9**. Comparison of R_e_ between contact tips and eutectic (EGaIn) contact.

**
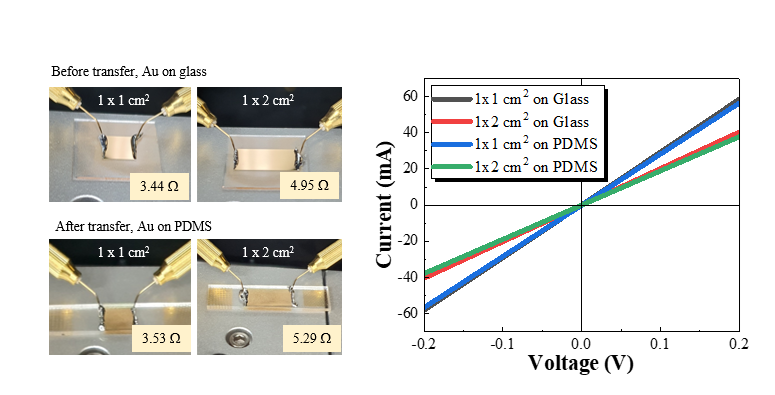
**

**Figure S10**. Comparison of R_e_ of Au on glass right after thermal deposition and MACE on PDMS. All thickness of Au was 30 nm.

**
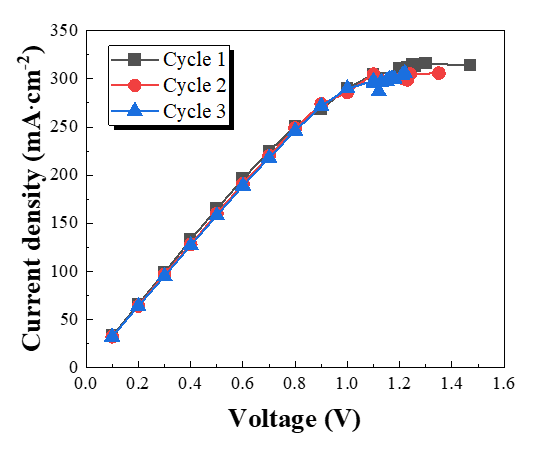
**

**Figure S11**. The current density behavior against voltage for MACE, size of which was 1 cm^2^, and thickness of Au was 30 nm. Maximum current density was saturated around 300 mA∙cm^- 2^.


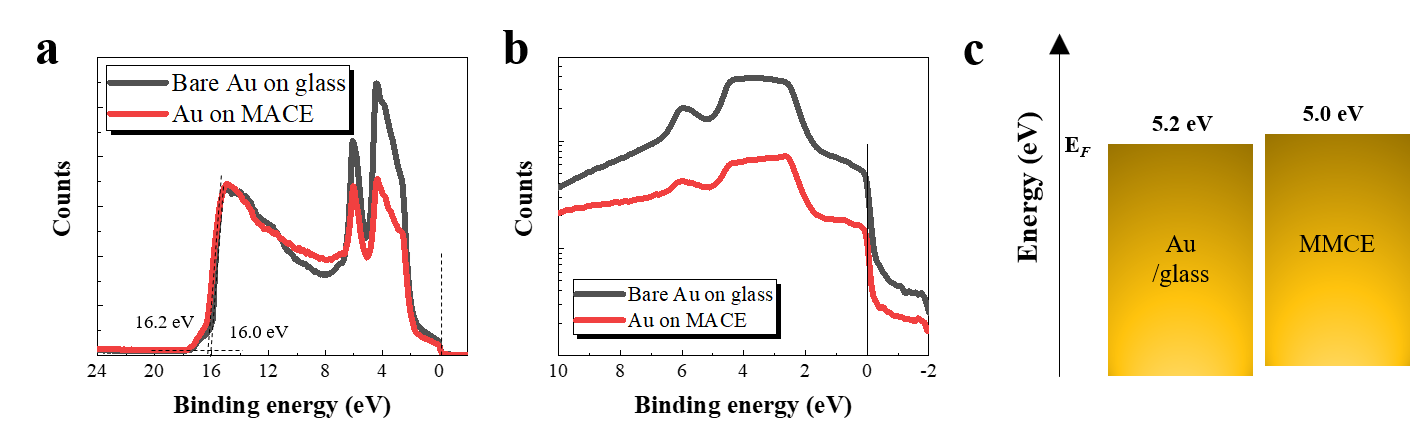


**Figure S12**. UPS profiles of bare Au film and MACE. a. for secondary electron cut-off. b. for Fermi level. c. Calculated work functions of bare Au and MACE by an equation of WF=21.2 eV−|SECO-Fermi level|.


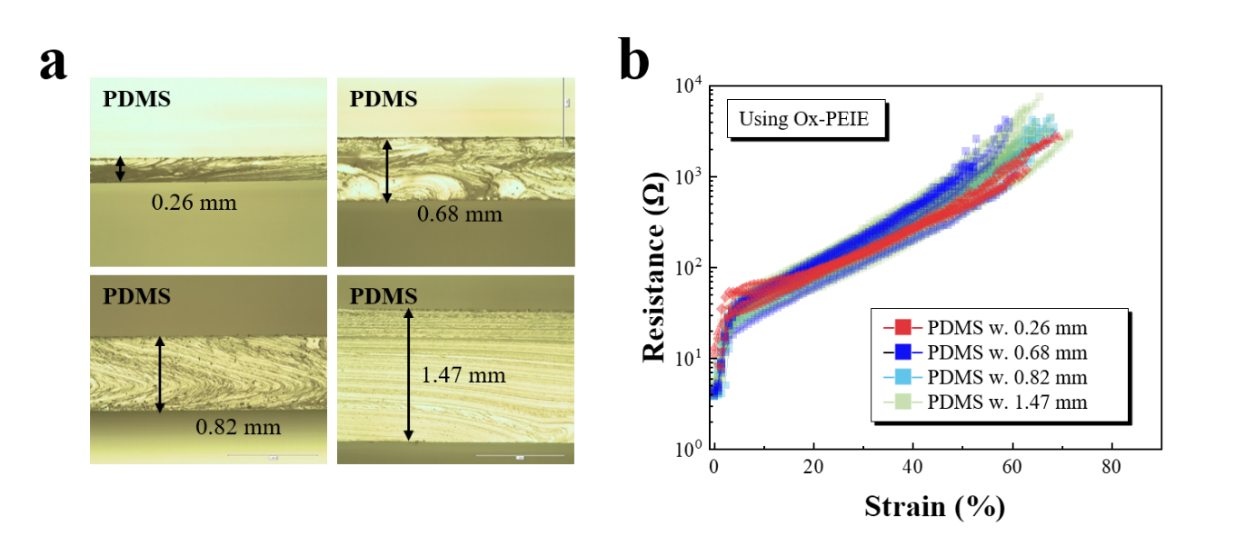


**Figure S13**. a. Optical images of cross-section of MACEs with various thickness of PDMS. Each thickness of PDMS was marked at inner image. b. Strain-resistance behavior of MACEs as the function of thickness of PDMS.


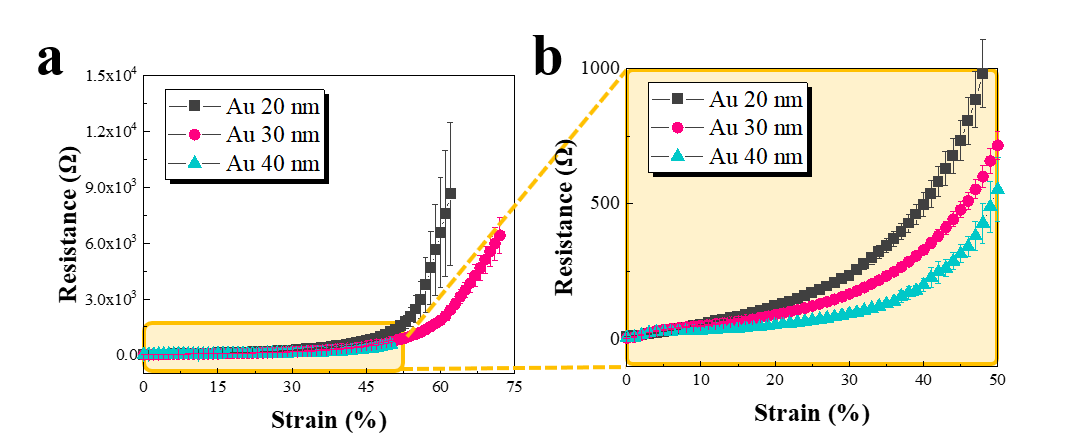


**Figure S14**. Strain-resistance behavior of MACEs with various Au thicknesses: 20, 30, and 40 nm. a. Overall results of MACEs as a function of strain. b. Enlarged view of the highlighted yellow region in (a). All samples were evaluated up to their critical strain. Over five samples were tested under each condition.


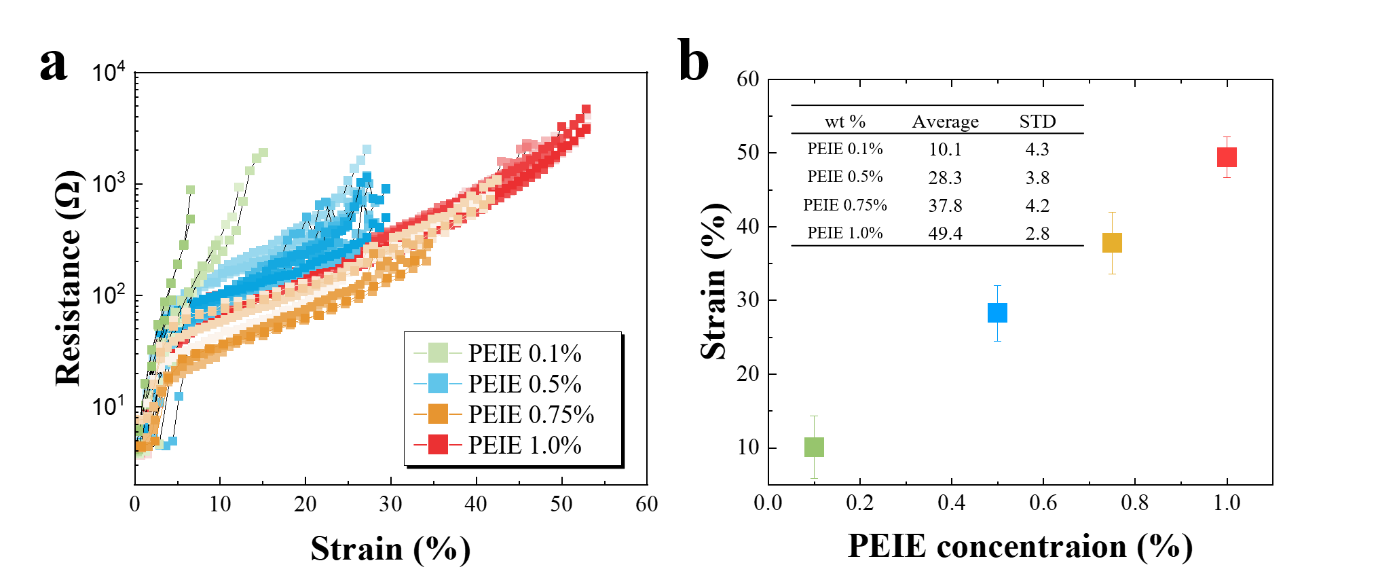


**Figure S15**. a. Strain-resistance behavior of MACEs processed by the various concentrations of bare PEIE. b. Strain-PEIE concentration plot. Average critical stain and standard deviation (STD) values were recorded in the inset.

**Table S1**. Resistance changes of MACE fabricated using bare and Ox-PEIE as a function of strain.

| Bare PEIE | | | | | Ox-PEIE | | | | |
| --- | --- | --- | --- | --- | --- | --- | --- | --- | --- |
|  | Stretching | | Releasing | |  | Stretching | | Releasing | |
| Strain  (%) | Re avg.  (Ω) | Standard deviation (±) | Re avg.  (Ω) | Standard deviation (±) | Strain  (%) | Re avg.  (Ω) | Standard deviation (±) | Re avg.  (Ω) | Standard deviation (±) |
| 0.0 | 4.1 | 0.1 | 4.1 | 0.1 | 0.0 | 4.2 | 0.8 | 4.2 | 1.1 |
| 1.8 | 11.9 | 1.2 | 10.6 | 1.2 | 2.5 | 21.4 | 2.5 | 20.4 | 1.8 |
| 3.7 | 32.1 | 1.8 | 33.0 | 3.6 | 4.9 | 34.0 | 2.1 | 38.2 | 2.2 |
| 5.5 | 48.4 | 2.8 | 49.2 | 4.7 | 7.4 | 41.6 | 2.4 | 46.2 | 2.5 |
| 7.3 | 59.0 | 3.0 | 60.5 | 5.4 | 9.9 | 48.7 | 2.7 | 52.6 | 2.7 |
| 9.1 | 69.9 | 3.6 | 69.6 | 6.0 | 12.3 | 57.4 | 3.1 | 59.1 | 3.1 |
| 11.0 | 80.6 | 4.1 | 78.8 | 6.2 | 14.8 | 67.0 | 3.9 | 66.7 | 3.6 |
| 12.8 | 92.7 | 4.6 | 88.8 | 6.2 | 17.3 | 79.1 | 4.5 | 75.3 | 4.1 |
| 14.6 | 106.1 | 5.0 | 99.2 | 6.3 | 19.7 | 94.0 | 5.3 | 85.5 | 4.8 |
| 16.4 | 122.4 | 5.5 | 111.6 | 6.7 | 22.2 | 110.0 | 6.1 | 97.4 | 5.7 |
| 18.3 | 140.2 | 6.3 | 127.6 | 7.0 | 24.7 | 129.7 | 7.5 | 111.6 | 6.6 |
| 20.1 | 160.8 | 7.3 | 142.9 | 7.0 | 27.1 | 151.7 | 9.1 | 128.8 | 8.3 |
| 21.9 | 183.4 | 7.8 | 161.6 | 7.6 | 29.6 | 177.9 | 11.3 | 149.4 | 9.9 |
| 23.8 | 210.9 | 9.0 | 183.0 | 8.0 | 32.1 | 211.7 | 14.5 | 174.3 | 12.1 |
| 25.6 | 243.1 | 9.7 | 207.9 | 9.1 | 34.5 | 247.4 | 17.2 | 205.9 | 14.8 |
| 27.4 | 281.5 | 11.0 | 237.5 | 10.7 | 37.0 | 291.3 | 20.7 | 243.2 | 19.0 |
| 29.2 | 323.4 | 12.0 | 270.1 | 13.1 | 39.5 | 346.7 | 24.9 | 289.3 | 23.1 |
| 31.1 | 384.9 | 16.3 | 309.9 | 15.1 | 41.9 | 414.3 | 31.5 | 347.0 | 29.5 |
| 32.9 | 446.1 | 18.7 | 358.1 | 17.4 | 44.4 | 499.2 | 41.6 | 416.5 | 36.1 |
| 34.7 | 525.7 | 24.6 | 419.9 | 20.3 | 46.9 | 591.0 | 51.2 | 505.0 | 44.9 |
| 36.5 | 615.8 | 31.5 | 490.0 | 25.6 | 49.3 | 732.4 | 74.2 | 619.4 | 58.8 |
| 38.4 | 726.0 | 36.2 | 574.1 | 28.7 | 51.8 | 903.9 | 94.2 | 778.8 | 87.8 |
| 40.2 | 884.2 | 50.4 | 677.1 | 37.5 | 54.3 | 1139.0 | 131.9 | 961.2 | 115.1 |
| 42.0 | 1053.6 | 57.2 | 824.1 | 43.5 | 56.7 | 1436.1 | 162.1 | 1213.6 | 150.1 |
| 43.8 | 1320.2 | 74.9 | 1040.0 | 77.2 | 59.2 | 1874.8 | 228.9 | 1565.5 | 203.2 |
| 45.7 | 1649.9 | 111.2 | 1443.0 | 225.4 | 61.7 | 2512.7 | 362.7 | 2100.1 | 304.3 |
| 47.5 | 2040.9 | 189.1 | 1940.0 | 379.8 | 64.1 | 3373.5 | 509.8 | 2989.0 | 551.9 |
| 49.3 | 2574.8 | 288.8 | 2500.7 | 526.2 | 66.6 | 4339.9 | 694.1 | 3868.4 | 845.0 |
| 51.2 | 3097.2 | 410.0 | 3160.9 | 654.8 | 69.1 | 5476.0 | 946.5 | 4811.0 | 1139.9 |
| 53.0 | 3747.4 | 561.4 | 3913.7 | 774.1 | 71.5 | 6659.9 | 1234.8 | 5763.9 | 1442.3 |


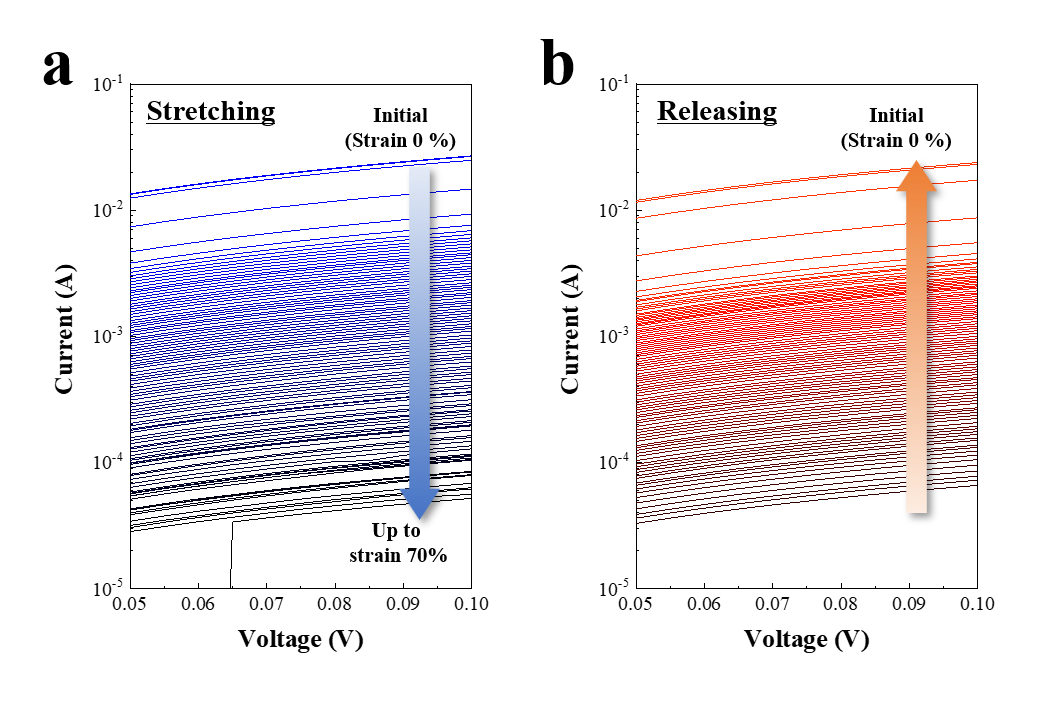


**Figure S16**. *I-V* characteristics of the best MACE by stretching and releasing tensile strain. a. when stretching up to the critical strain, which was 70 % and b. when releasing from the critical strain to the initial status (strain 0%). The MACE was fabricated using a 30 nm thickness of Au and Ox-PEIE.

**Comparison in electrical and stretchability with previous metal-based stretchable electrodes**

Evaluating the stretchability and electrical properties of stretchable electrodes is essential for comparing various candidates. However, despite significant attention toward next-generation electrodes, the establishment of comprehensive and standardized parameters and protocols remains ambiguous. The lack of uniformity in evaluation creates confusion even within the same category, as specific terms such as conductivity (S∙cm⁻¹), sheet resistance (R_sh_, Ω∙sq⁻¹), and electrical resistance (R_e_, Ω) have been used inconsistently across different studies. This makes it difficult to compare the electrical properties of previous works. In this work, we compared the conductance of metal-based electrodes, and we used the measured R_e_ and R_sh_ estimated from evaluation instead of conductivity, which does not include a thickness term, to avoid gaps with the conductance of practical electrodes. Figure S17 shows the initial R_sh_ and stretchability of metal-based electrodes, noting the corresponding thickness of the conductive metal layer as reported in previous studies next to their labels. For detailed information, we have summarized by sorting by the types of metal-based electrodes, and noting the thickness, and elastic substrate, as well as the stretchable method in Table S2.

In solid metal-based stretchable electrodes, to achieve stretchability, pre-stretching of the elastic substrate has been widely adopted.^[11–13]^ In this approach, the elastomer substrate is pre-stretched before the deposition of conductive materials on it, and after the formation of the conduction layer, the applied strain is released, causing the template to shrink and resulting in a buckle-and-fold structure. This process allows the pre-stretched electrode to maintain stable stretchability to the level it was initially stretched before the conductive layer was deposited.^[13]^ However, this method strongly relies on the physical transformation of the substrate, making it challenging to address the fundamental issues of stretchability. Alternatively, strategies for the morphological modification of metal into the metal mesh that consider stress propagation or a metal wire that secures the electron pathways along fibrils are intensely investigated for stretchable electrodes. Through these efforts, the mechanical stretchability of these classes of electrodes showed over 50%. In recent work based on the Ag wire, the highest stretchability was achieved up to 250% for free-standing and up to 1000% with the assistance of an ultrathin elastic template.^[14]^

Our stretchable electrode (MACE), based on a solid metal film, resisted tensile strain up to 70%. This result exceeds the stretchability of human skin, which is approximately 30%. It is worth noting that this is the best result achieved without the physical transformation of an elastic template. When compared to the best results achieved by metal wire electrodes, our results showed relatively low performance. However, when considering critical points such as the thin layer (tens of nanometer thickness), the absence of organic components hindering charge transfer between wires, and the use of a vapor deposition process that is not reliant on synthesis, we believe that MACE has sufficient potential for wide-ranging applications.


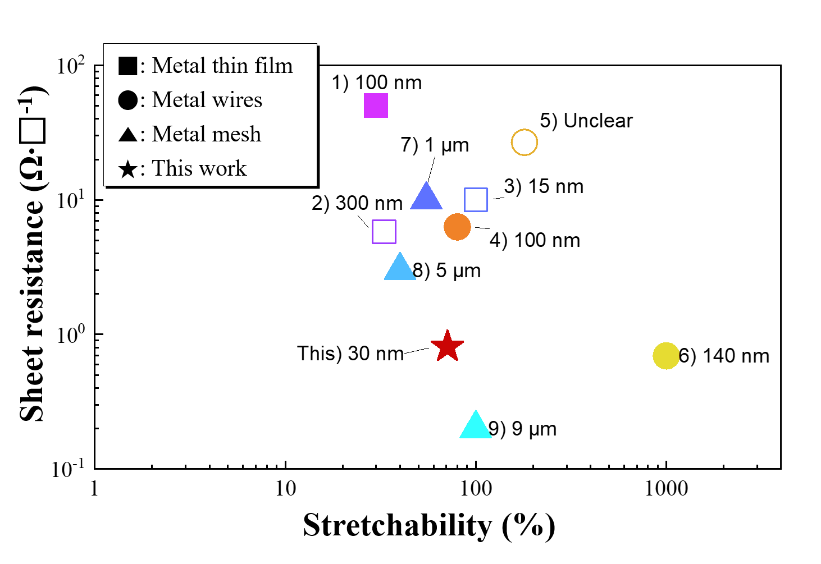


**Figure S17**. Comparison of MACE with other stretchable electrodes for maximum stretchability and sheet resistance. Metal electrodes are categorized by the type of metal. An empty symbol indicates that the electrode's stretchability was achieved through pre-stretching the substrate. The R_sh_ and stretchability means initial resistance before stretching and the maximum capability, respectively.

**Table S2**. Summary of stretchable metal based-electrode and organic-based electrode.

| Label | Group | Materials  (thickness) | Strategy | Substrate | Max.  Stretchability | Resistance  (Ω, Ω∙sq^-1^) | Remark |
| --- | --- | --- | --- | --- | --- | --- | --- |
| - | Thin film | Au  (20 nm) | Metal-amine Coordination bonding | PDMS | 60% | 14.0 Ω | This  work |
| - | Thin film | Au  (30 nm) | Metal-amine Coordination bonding | PDMS | 70% | 4.0 Ω  (0.813 Ω∙sq^-1^) | This  work |
| - | Thin film | Au  (40 nm) | Metal-amine Coordination bonding | PDMS | 48% | 3.7 Ω | This  work |
| 1 | Thin film | Au  (100 nm) | Nanomesh substrate | PU | 30% | 50 Ω | ^[15]^ |
| 2 | Thin film | Ag  (15 nm) | PDMS pre-stretching | PDMS | 100% | 5.8-6.9 Ω | ^[11]^ |
| 3 | Thin film | Ag  (300 nm) | PDMS pre-stretching | PDMS | 35% | 69 Ω∙sq^-1^ | ^[13]^ |
| 4 | Metal wires | Ag wires  (100 nm) | Metal repelling layer | PET,  PDMS | 80% | 6.3 Ω∙sq^-1^ | ^[16]^ |
| 5 | Metal wires | Ag wires  (Diameter: 27 nm) | PDMS pre-stretching, post solvent treatment for wire buckling | PDMS | 50% | 26.8 Ω∙sq^-1^ | ^[12]^ |
| 6 | Metal wires | Ag wires  (140 nm) | Ag wire stacking  Nanomembrane | SEBS | 1000% | 103,100 S/cm  (0.693 Ω∙sq^-1^) | ^[14]^ |
| 7 | Metal mesh | Cu  (1 μm) | Photolithography, Electrodeposition | PDMS | 55% | 2 Ω∙sq^-1^ | ^[17]^ |
| 8 | Metal mesh | Ag wire mesh  (5 μm) | Woven metal mesh using Ag wires | PDMS | 40% | 3.2 Ω∙sq^-1^ | ^[18]^ |
| 9 | Metal mesh | Ag NP, Cu  (9 μm) | Ag NPs spreading and Cu electroplating | PDMS | 100% | 0.15 Ω∙sq^-1^ | ^[19]^ |

* Abbreviation of the substrates follows next to chemical full name; polyurethane (PU), polyethylene terephthalate (PET), poly(styrene-ethylene-butylene-styrene) (SEBS), thermoplastic polyurethane (TPU), poly(styrene-block-butadiene-block-styrene) (SBS), and hydrogel copolymers using poly(ethylene glycol) diacrylate and 2-hydroxyethyl acrylate.

* Electrical resistance (R_e_, Ω) or sheet resistance (R_sh_, Ω∙sq^-1^) were used depending on relevant works. In our work of MACE using Au 30 nm, R_sh_ was estimated from the R_e_ showing identical R_e_ compared to bare Au film, and it was recorded in parentheses.

* If the conductivity was preferred in the reference work, conductivity was transformed to R_sh_ using providing thickness and conductivity, and it was recorded in parentheses.


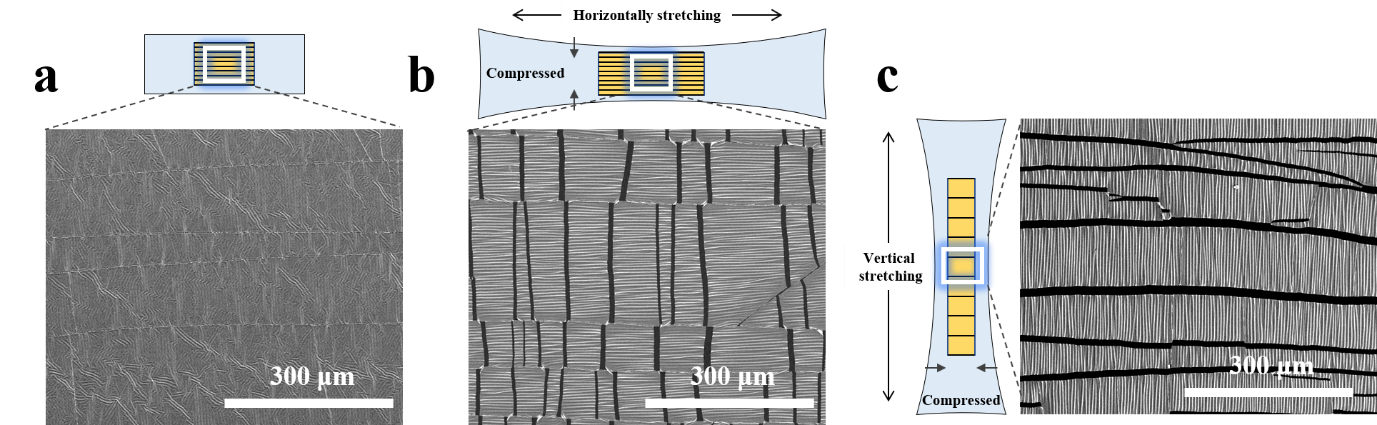


**Figure** **S18**. SEM images of MACE in different stretched states: a. without stretching, b. horizontally stretched, and c. vertically stretched. In the stretched cases, MACE was subjected to a 30% strain in both horizontal and vertical directions.

**
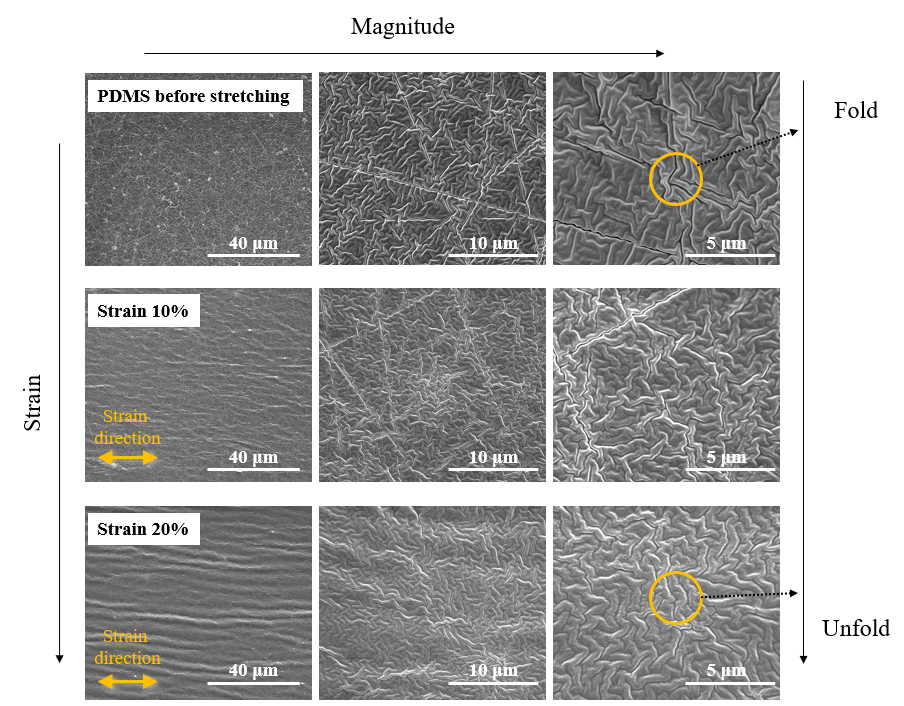
**

**Figure S19**. SEM images of bare PDMS depending on the strain. The PDMS was stretched to the horizontal waymarked in yellow.


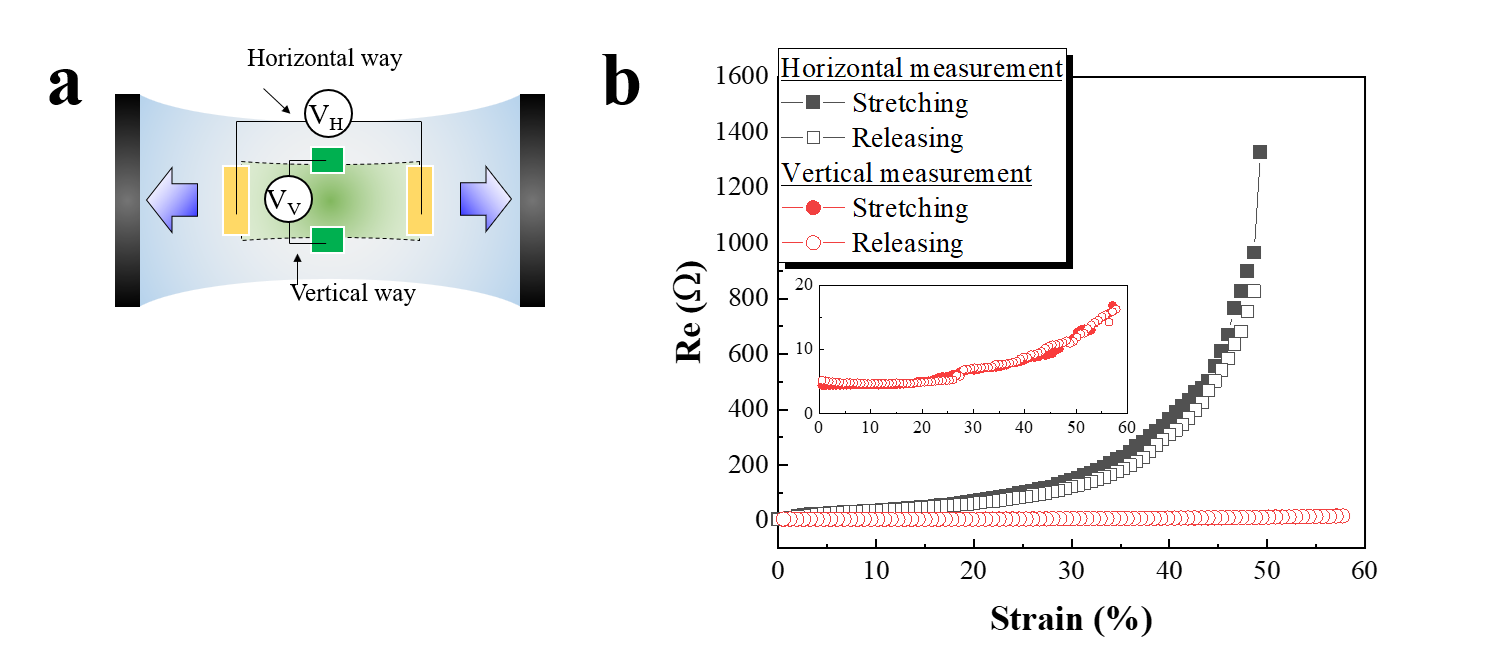


**Figure S20**. a. Illustration of horizontal and vertical resistance (Rₑ) measurements of MACE under strain. b. Rₑ as a function of strain, showing filled symbols for measurements taken during stretching and empty symbols for measurements taken during release after reaching critical strain. MACE was fabricated using Ox-PEIE.


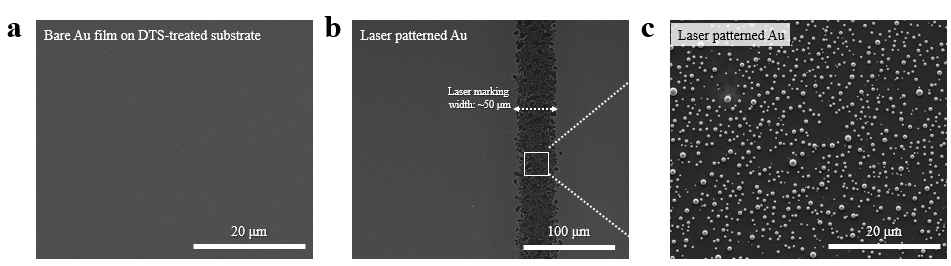


**Figure S21**. SEM images after laser ablation to Au 30 nm thickness film. a. Bare Au surface on DTS-treated substrate. b. Surface morphology after laser marking, forming a line, and line width was approximately 50 μm. c. High-magnification image of laser patterned area, showing sub-micro size of Au particles.

**Surface oxidation after laser processing and enhancement adhesion with PEIE**

To explore the changes in surface functionality of Si depending on treatments, the contact angle measurement was conducted by dropping a water droplet on the bare Si wafer, DTS-treated Si (DTS-Si), and oxidized DTS-Si that was laser-illuminated, respectively. On a bare Si substrate, the water droplet spread well, and the specific angle could not be detected. In contrast, the DTS-Si showed a high contact angle of 92^o^, indicating that the surface functionality changes to hydrophobicity. After laser illumination on the DTS-Si, the contact angle reduced to approximately 32^o^, showing that the surface became hydrophilic again (Figure S22). These observations confirmed that the Si surface was modulated by treatments, and DTS-Si was turned to hydrophilicity after laser processing.

For further in-depth study, the surface bonding of Si was explored by XPS measurements, depending on the surface oxidation and PEIE deposition. Si 2p analysis showed remarkable changes in DTS-Si after laser illumination and PEIE deposition on laser-treated Si (Figure S23). In the case of DTS-Si, bonds of O-Si-O, Si-O, and Si-C were observed at 103.8, 103.1, and 102.5 eV, respectively (Figure S23a). These observations are identical to our previous work.^[20]^ After laser illumination, the proportion of O-Si-O increased, reducing the fraction of the Si-C peak. This indicates that the DTS surface was oxidized, which is consistent with the observations from the changes in contact angle (Figure S23b). When the PEIE layer was formed on this surface, a Si-N bond appeared at 101.9 eV,^[21]^ indicating relatively strong bonding (Figure S23c). This enhanced bonding likely results from the change of the Si surface functionality due to the laser treatment, coupled with an annealing process that promoted better adhesion with amine groups on the oxidized Si surface. Thus, we confirmed that an oxidized surface was formed on DTS- Si through laser illumination, and these areas exhibited improved adhesion with the PEIE layer.


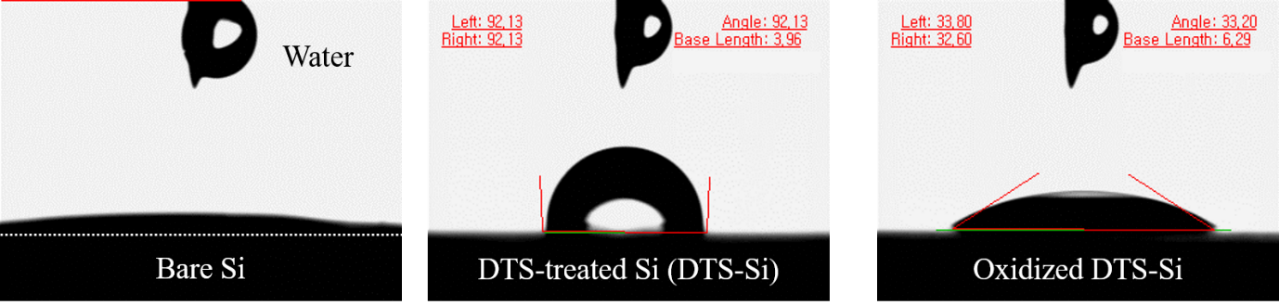


**Figure S22**. Contact angle depending on the surface treatment. a. bare Si with native oxide, b. DTS-treated Si, c. Oxidized DTS-Si after the laser treatment.


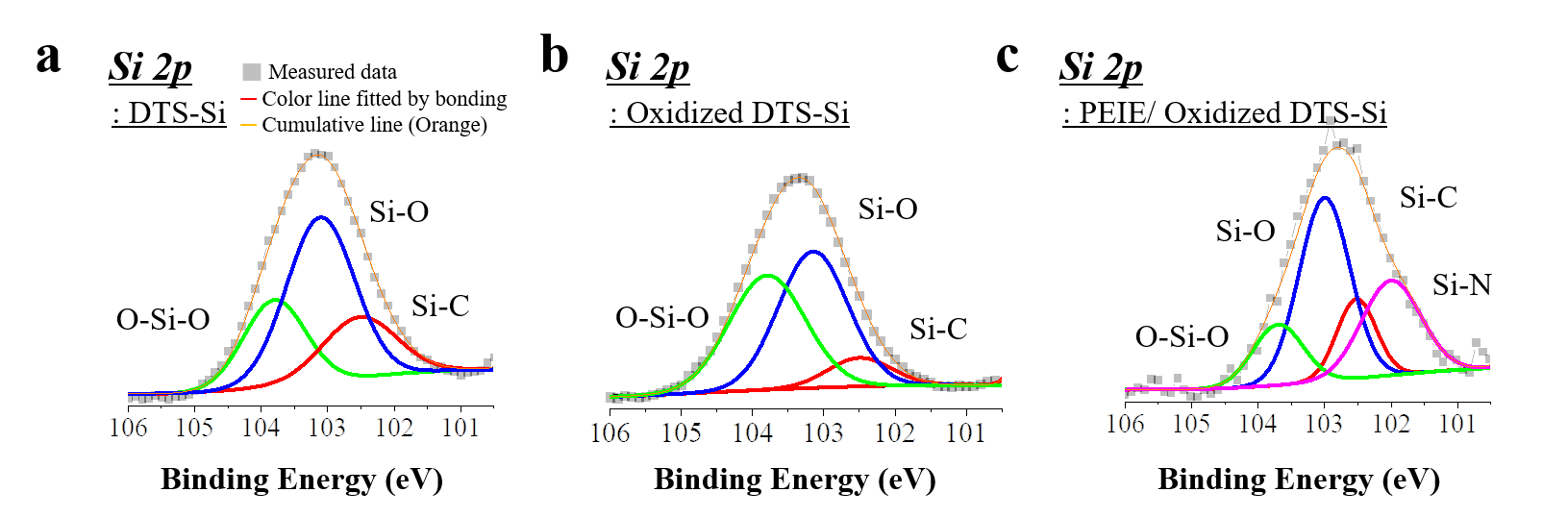


**Figure S23**. Si 2p XPS analysis of a. DTS-treated Si (DTS-Si), b. Oxidized DTS-Si, and c. PEIE/Oxidized DTS-Si by the laser illumination. The gray box represents the measured data, and the color lines represent each bond, except for the orange line, which represents the cumulative line.

**XPS analysis of Au particles depending on the presence of PEIE**

To investigate the interaction of Au particles and PEIE polymer, we prepared Au particle by laser processing across the entire Au film under ambient air conditions. It is worth noting that the laser treatment generates heat facilitating the surface oxidation of the DTS-treated surface and aggregates of the Au film into particles, increasing the total surface area of Au.

The Au 4f analysis confirmed that the position of main corresponding to the bulk, surface, and oxide were identical to those of Au film, however, the proportion of oxide and surface component become increased compared with the result of pure Au film in Figure S8 (Figure S24a). Subsequently, when PEIE was deposited upon Au particles, the peaks shifted to lower energy in same manners. Notably, the surface peaks were significantly enhanced and shifted by far away from 1.37 eV (red dot-line), showing the clear shoulder peaks at 82 and 86 eV, respectively (Figure S24b). This indicates the formation of coordination bonding between the Au particles and the amine groups, with a bond strength stronger than that observed in the case of the Au film. Thus, we conclude that a strong interaction between Au and PEIE is established, particularly at the interface. Based on the observations and silicon analysis results shown in Figure S23, it is deduced that the PEIE chain interacts with both oxidized silicon and gold surfaces, serving as a web to anchor gold particles to the oxidized substrate.


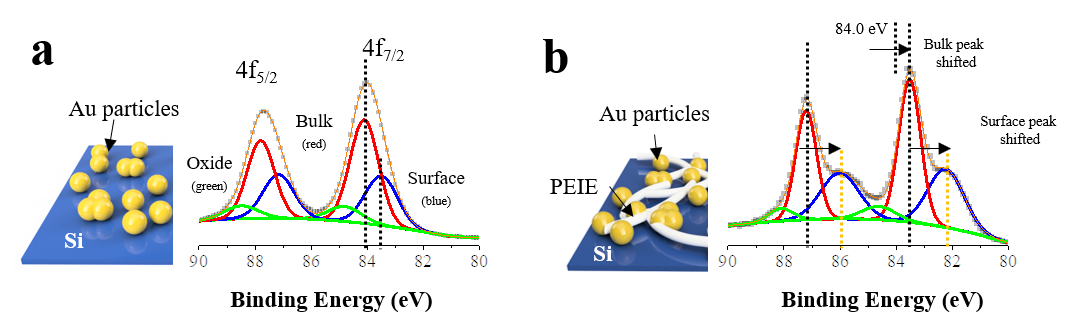


**Figure S24**. Au 4f XPS analysis for Au PR depending on the presence of the PEIE. a. Au particles, and b. Au particles covered by the PEIE layer. The gray square represents the measured data; each color line corresponds to a specific bonding: red for bulk, blue for surface, and green for oxide formation of Au. The orange line represents the cumulative line.

**Transmittance spectra of Au particle film depending processes**

To examine the effect of transferred residues on optical properties, the transmittance of the Au film was measured throughout each sequential process (Figure S25). When the Au film (yellow) turned into Au particles (blue), the average visible transmittance changed from 30.8% to approximately 80% due to the formation of empty spaces by the aggregation. When the PEIE layer was formed on the Au particle film, the transmittance spectra of Au particles/PEIE (green) showed a minor change. For the Au particle film, the exfoliation process was also executed in the same manner, but it was not transferred and left as a residue. The residue film showed the same transmittance spectrum (green dot) as that before exfoliation (green). Furthermore, the spectrum of the area where PDMS was formed on the Au particle film (blue dot) was identical to bare PDMS, indicating that few Au particles transferred to the PDMS side, and their effect was so minor that it could be considered negligible. Thus, we concluded that the transfer of Au particles was significantly prohibited under the optimized condition and did not induce any notable change in transmittance.


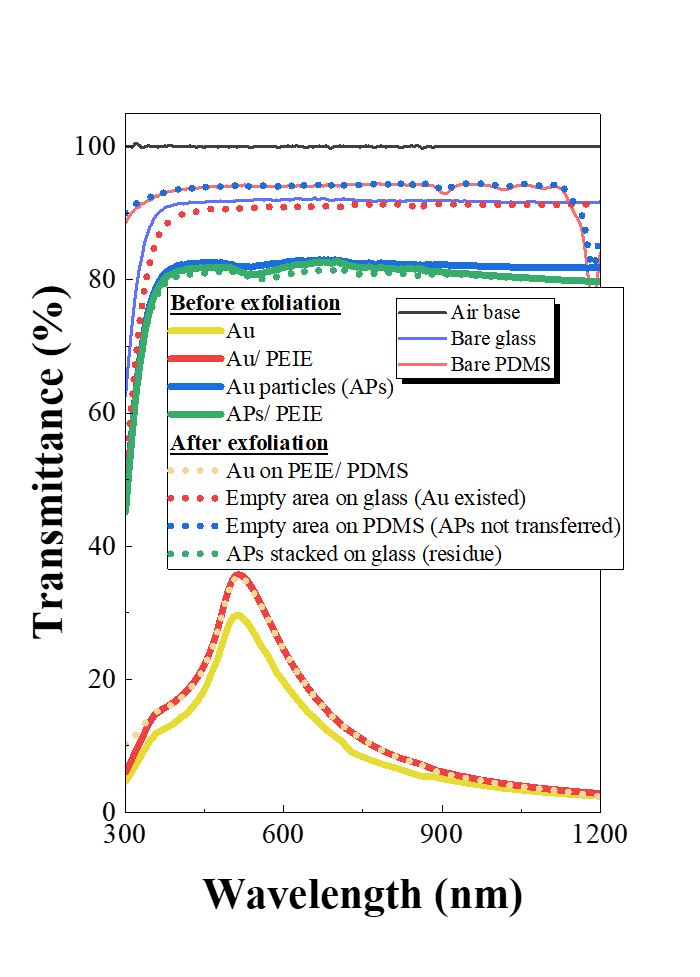


**Figure S25**. Transmittance spectra of Au particle film formed by laser patterning and its changes depending on the subsequent processes of PEIE coating and exfoliation.

**Morphology observation of Au film after laser patterning and before and after exfoliation**

SEM images in Figure S26 reveal morphological changes of the Au film after laser treatment, the residue film on the substrate, and the transferred MACE on PDMS after exfoliation. After patterning, the patterned and unpatterned areas were specified, and the boundary was clearly defined, with micro-sized Au particles observed in the high-magnification image (Figure S26a). Following the sequential procedure and exfoliation process, the unpatterned regions containing the Au film were transferred, leaving the substrate bare, while Au particles remained stacked on the residual film even after exfoliation. Additionally, in high-magnification images, some Au flakes were observed. This results from the patterning boundary not being narrowly defined at a scale of tens of micrometers due to heat propagation during nanosecond laser processing (Figure S26b). In contrast, on the side of the patterned MACE, the transferred Au film exhibited typical wrinkles and buckles. At high magnification, a few transferred particles were observed (Figure S26c).

For further analysis, the patterned area was assessed after the exfoliation process using energy-dispersive X-ray (EDX) spectroscopy, which profiles depths of several micrometers. The patterned regions containing Au particles and film primarily revealed elements of Si, O, and Au (Figure S27a), with Si observed throughout the area and O predominantly detected in the laser-patterned regions, confirming localized surface oxidation. Au analysis indicated that the particles were aggregated from the Au film. In the patterned Au transferred on the PDMS side after exfoliation, some stains involving Au particles were detected (Figure S27b). EDX analysis verified that not all stains were Au particles; some resulted from the surface morphology of the PEIE/PDMS template. Additionally, the EDX mapping showed N distributed across the entire area, strongly indicating polymer separation after exfoliation.


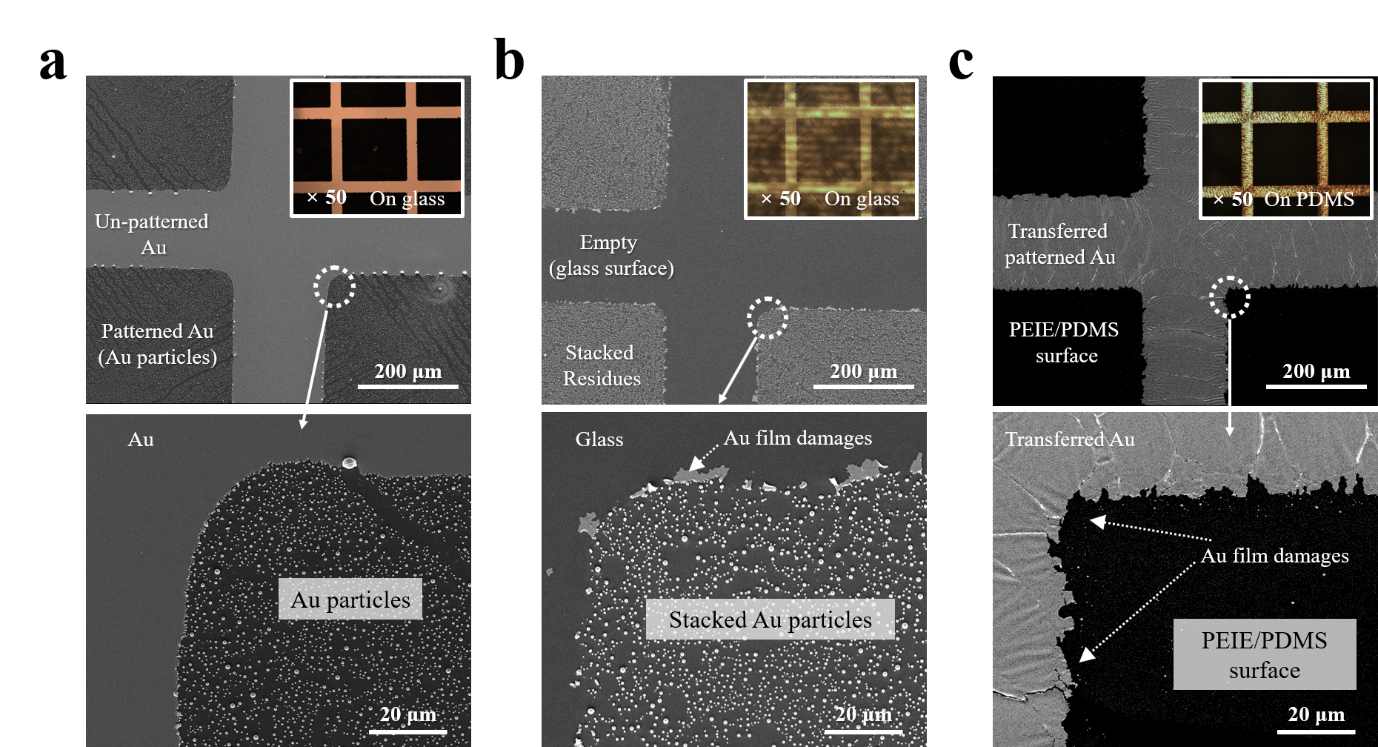


**Figure S26**. SEM images after laser patterning and residue and transferred patterned MACE after exfoliation. a. mesh patterned Au film and b. and c. residue film and transferred patterned MACE after exfoliation. Inset is OM image correlated with SEM sample.


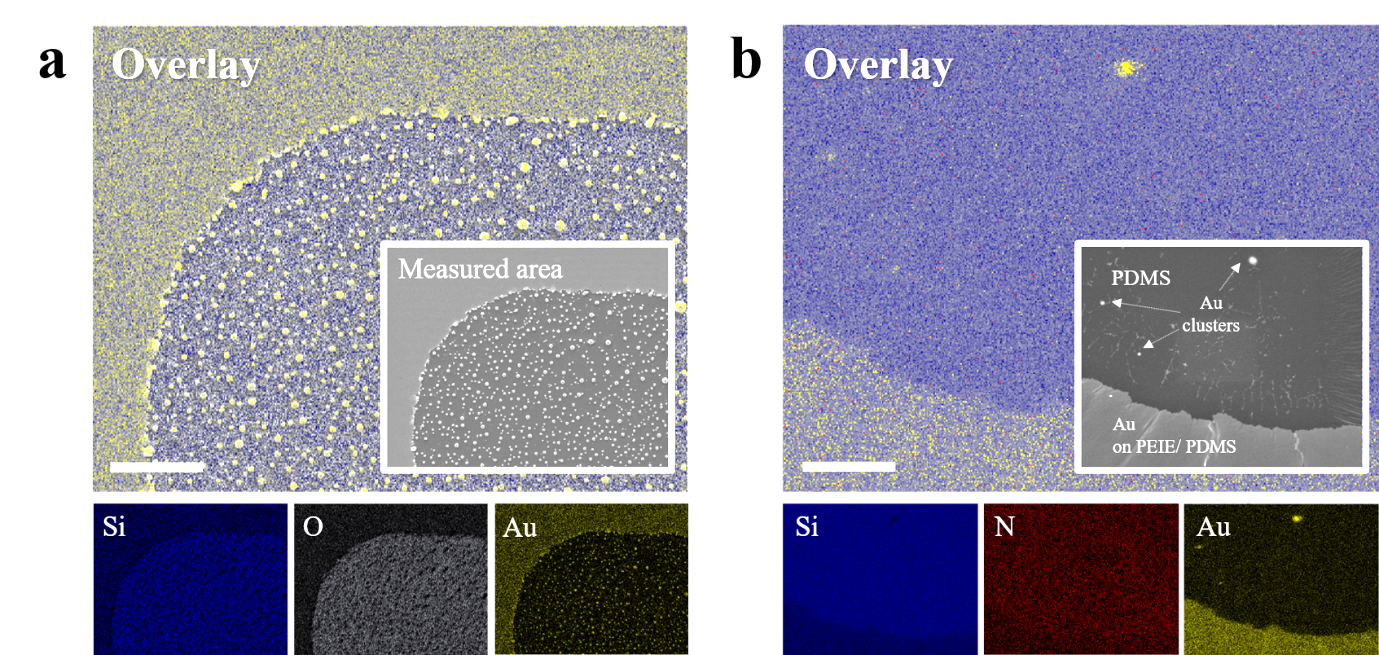


**Figure S27**. EDX analysis of a. the boundary of Au by laser patterning. and b. transferred Au film on PDMS after exfoliation process. Si (blue), Oxygen (white), Au (yellow), and N (red). Scale bar is 5 μm.


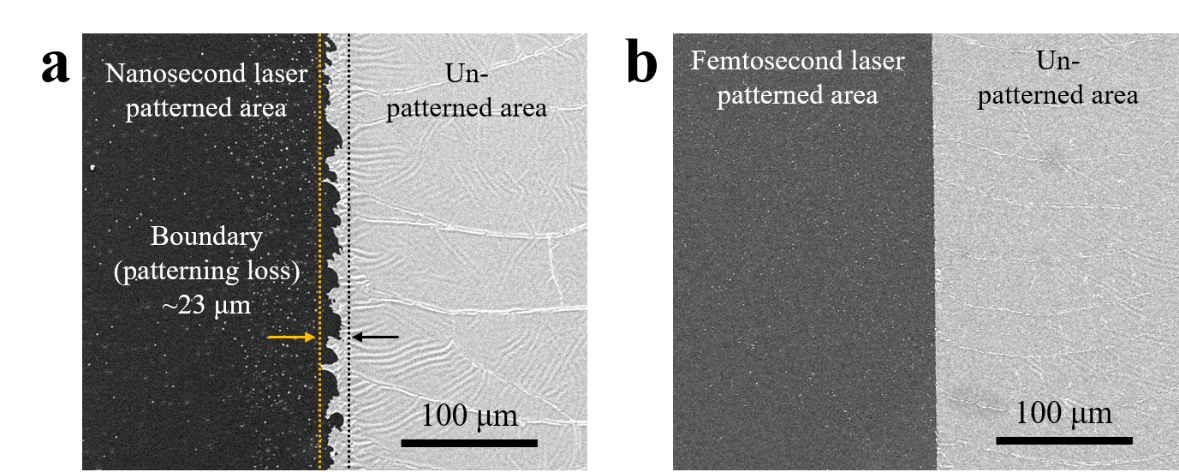


**Figure S28**. SEM images of the patterned Au film processed by nanosecond (ns) and femtosecond (fs) lasers. a. for ns-laser-processed Au. b. for fs-laser-processed Au. Depending on the type of ns or fs laser sources, patterning loss was significantly different showing approximately 23 and 1 μm, respectively.


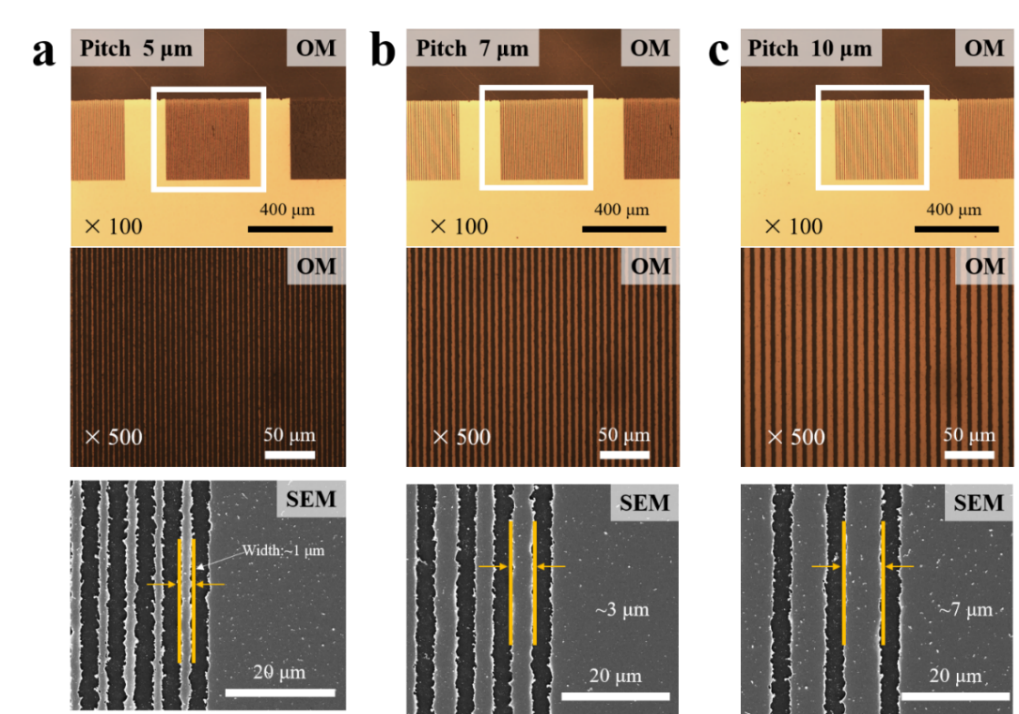


**Figure S29**. Optical and scanning electron microscopy images of Au film processed by femtosecond laser depending on the pitch. In this system, the diameter of the laser beam was around 2 μm. a. for pitch of 5 μm. b. for pitch of 7 μm. c. for pitch of 10 μm. Each linewidth was estimated to be approximately 1, 3, and 7 μm.


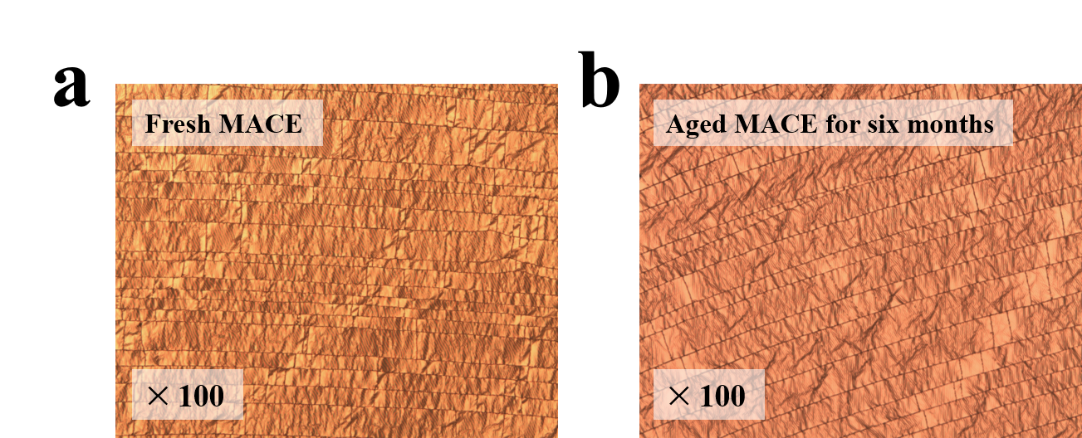


**Figure S30**. Optical images of (a). fresh MACE and (b). aged MACE, which has been stored in ambient air for six months.


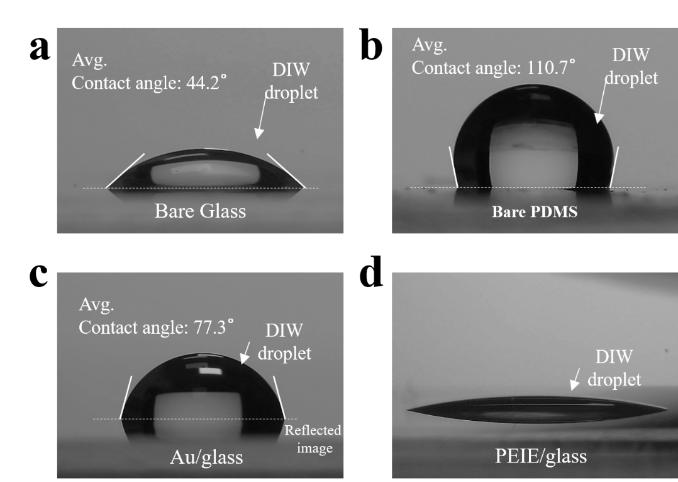


**Figure S31**. Contact angle images of (a). glass, (b). PDMS, (c). Au on glass, and (d). PEIE on glass. The contact angle was measured using a deionized water (DIW) droplet.

**Lamination technology of patterned MACE in photovoltaics**

As discussed, it was confirmed that the exposed Au of MACE is similar to that of a bare Au film. These findings suggest that the exposed Au area of MACE can form additional coordination bonds with an underlying layer consisting of atoms such as O, N, and S, and indicate that p-MACE can be deposited by lamination, thereby serving as the grid electrode for efficiently transferring carriers.^[22,23]^

To verify our hypothesis, we directly laminated MACE on Poly(3,4-ethylenedioxythiophene) polystyrene sulfonate (PEDOT:PSS), which contains many O and S atoms in chemical structure, and this layer is a representative functional layer in photovoltaics (PVs) based on perovskite, organic, and hybrid PV. Thereafter, when MACE was delaminated from PEDOT:PSS, residual traces were clearly observed across the PEDOT:PSS surface, and XPS analysis confirmed that these residues were Au, suggesting that strong interfacial interactions can form even through lamination (Figure S32).

To apply the p-MACE to PV devices, the h-PV and were separately prepared. For PVs, Si hybrid PVs (h-PVs) with an n-i-p structure was adopted in our previous work ^[24]^. The h-PVs were established using n-Si (1−10 Ω·cm, CZ grown), the size of which was 2 cm × 2 cm. To form PN junction, the native oxide on the surface of n-Si was removed by immersing it in buffered oxide etch (BOE, 6:1) for 1 minute. Subsequently, a stock PEDOT:PSS solution was deposited on n-Si using spin-coating method at 1000 rpm for 45 seconds, and annealed at 150 °C for 20 minutes. Next, a 50 nm layer of Au was deposited as the top electrode for collecting photogenerated carriers, with a metal shadow mask placed on the top surface to define the active area. On the rear side, Al/Ag (100 nm/100 nm) was deposited to extract photoelectrons under high vacuum pressure (< 5 × 10⁻⁷ torr). For p-MACEs, we prepared two different p-MACEs consisting of simple square patterns measuring 1500 μm × 1500 μm, repeating across 1 cm², with the pattern spacing adjusted to 300 μm or 500 μm. The Au coverage area of each p-MACE was estimated to be 30.6% and 43.8%, respectively.

Without grid electrodes, as the active size of h-PV was increased from the optimized size of 25 mm² to 100 mm², the power conversion efficiency (PCE) decreased from approximately 11% to around 5%, emphasizing the importance of the grid electrode. This degradation mainly resulted from the severe drop in fill factor (FF) from 70.2% to 40.5%, while the changes in open-circuit voltage (V_oc_) and photocurrent density (J_sc_) were negligible (Figure S33a–b and Table S3).

To verify the feasibility of p-MACE as a grid electrode, we laminated p-MACE onto PEDOT:PSS of h-PV without the use of any adhesion-enhancing assistance (Figure S33c). After lamination, V_oc_ was slightly improved; this is likely due to alleviation of the charge accumulation by the grid electrode, and J_sc_ reduced proportionally to the covered area by Au area compared to before the introduction of p-MACE (Figure S33d–e). Meanwhile, the FF increased from 40.5% to 52.4% and 67.2%, respectively (Figure S33f). In conclusion, the PCE of p-MACE was improved compared to that of h-PV without a grid electrode (Table S3). This enhancement is attributed to the effective transport of photocarriers through the Au grid. Furthermore, when p-MACE was removed from the h-PV, residue from the grid lines was observed. This approach highlights the novel potential of MACE based on coordination bonding through the lamination method. We believe that this approach can be further advanced through sophisticated patterning design, maximizing photocarrier generation and effective collection.

**Analysis properties of PV**

The PV’s performance was characterized using a solar simulator (XES-301S/EL100, S34SAN-EI Electric Co., Ltd., Osaka, Japan) under 1sun (AM 1.5G) illumination (100 mW cm^−2^). To measure the external quantum efficiency (EQE), a measurement system (K3100 Spectral IPCE Measurement system (McScience) was used to analyze the incident photon-to-current conversion efficiency.


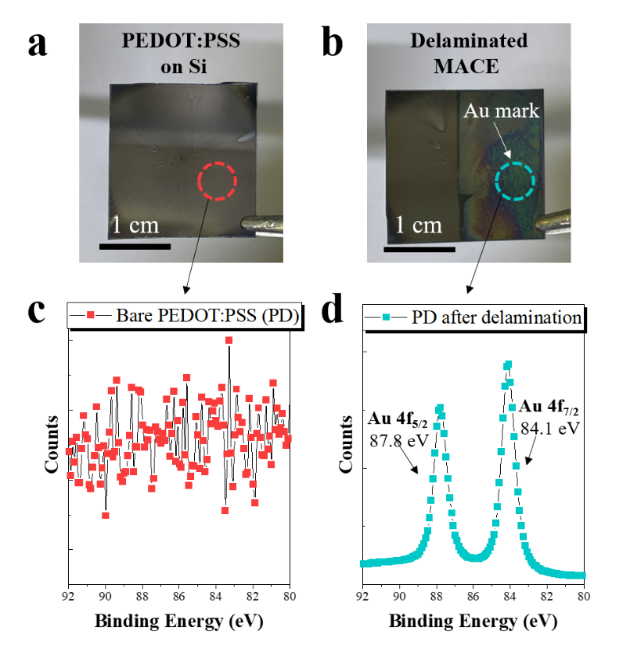


**Figure S32**. Photographs of a. the bare PEDOT:PSS film and b. PEDOT:PSS film where MACE was laminated and delaminated. PEDOT:PSS film was deposited on Si. XPS analysis for Au 4f. c. for bare PEDOT:PSS film and d. for residues on PEDOT:PSS film. When MACE was stamped on PEDOT:PSS film, it was half-covered to compare the difference by Au residue. XPS analysis confirmed the transferred Au was transferred after delamination.


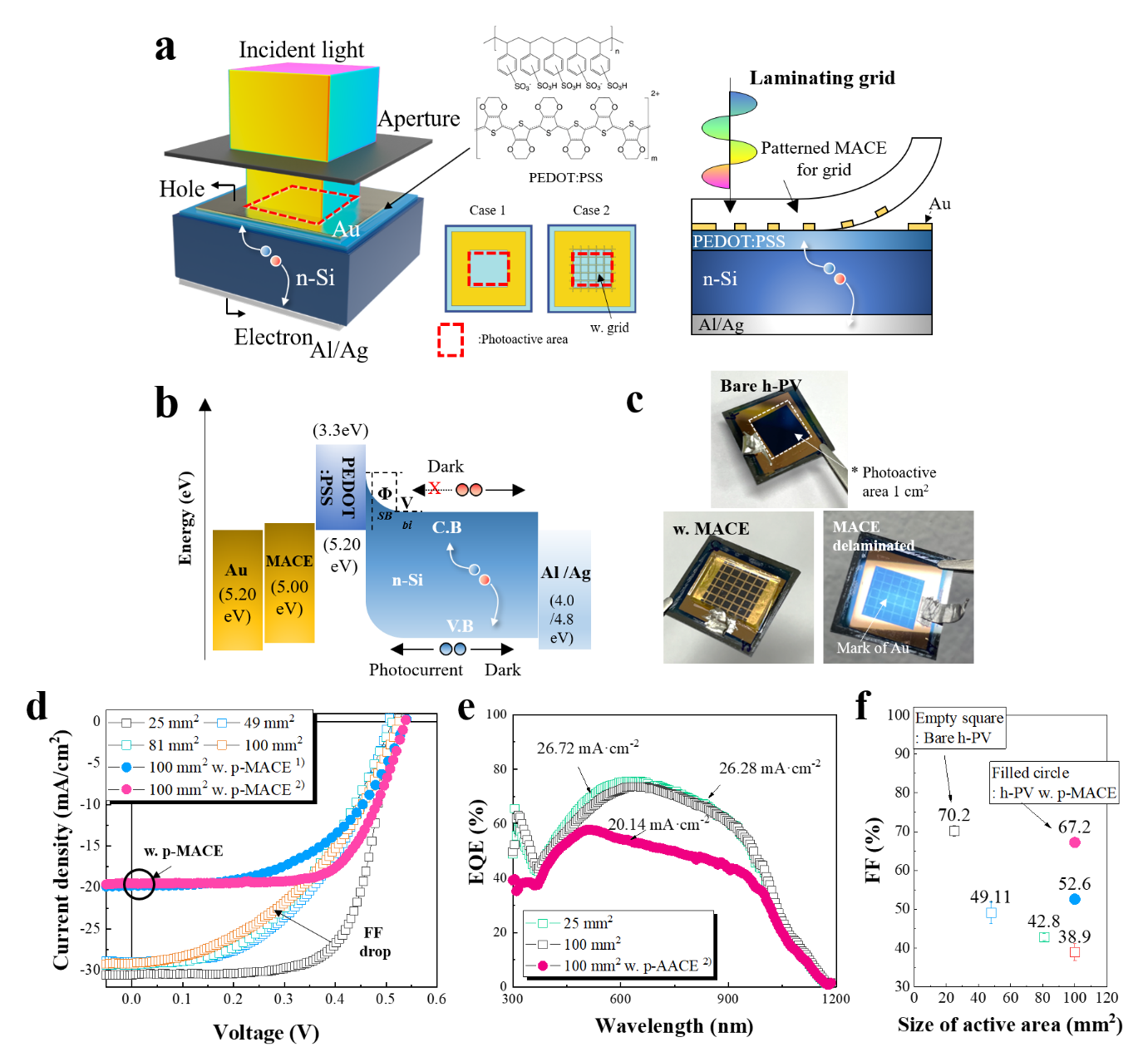


**Figure S33**. a. Illustration of organic hybrid Si photovoltaics (h-PV) based on PEDOT:PSS and grid electrode formation by laminating p-MACE. b. Energy diagram of h-PV. c. photographs of large-scale bare h-PV with 1cm^2^ and laminated h-PV using p-MACE and after MACE was delaminated. d. J-V curve of h-PV depending on the size of the active area and the presence of p-MACE. p-MACE were prepared by adjusting the linewidth while keeping the patterning area of 1500 μm × 1500 μm. The p-MACE ^1)^ and p-MACE ^2)^ consist of the width of 300 and 500 μm, respectively. The occupied Au area of each p-MACE was estimated to be 30.6% and 43.8%, respectively. e. External quantum efficiency (EQE) spectrum. f. fill factor (FF) changes of h-PV depending on active area and the presence of p-MACE.

**Table S3**. Photovoltaic parameters of h-PV depending on the size of the active area and the lamination of p-MACE.

| Size  of h-PV  (mm^2^) | Laminated electrode | V_oc_  (V) | Jsc (mA/cm^2^) | FF  (%) | PCE  (%) |
| --- | --- | --- | --- | --- | --- |
| 25 | - | 0.50±0.01 | 30.7±0.30 | 70.5±1.34 | 10.6±0.3 |
| 47 | - | 0.49±0.02 | 29.8±1.10 | 47.9±2.77 | 7.3±0.11 |
| 81 | - | 0.51±0.02 | 30.3±1.61 | 42.8±0.94 | 6.7±0.10 |
| 100 | - | 0.51±0.03 | 28.8±0.83 | 38.9±2.2 | 5.8±0.07 |
| 100 | w. p-MACE^1)^  with Au 30.6% | 0.54±0.002 | 19.9±0.20 | 52.6±0.35 | 5.7 ±0.11 |
| 100 | w. p-MACE^2)^  with Au 43.8% | 0.54±0.003 | 19.8±0.39 | 67.2±1.02 | 7.1±0.20 |

* p-MACE ^1)^ and p-MACE ^2)^ consist of patterned squares of 1500 μm × 1500 μm and different linewidth of 300 and 500 μm, respectively.

* Abbreviations of V_oc_, J_sc_, FF, and PCE stand for open circuit voltage, current density, fill factor, and power conversion efficiency in same order.

* The electrical characteristics were assessed using more than five devices.

**Reference**

[1] Y. Zhou, C. Fuentes-Hernandez, J. Shim, J. Meyer, A. J. Giordano, H. Li, P. Winget, T. Papadopoulos, H. Cheun, J. Kim, M. Fenoll, A. Dindar, W. Haske, E. Najafabadi, T. M. Khan, H. Sojoudi, S. Barlow, S. Graham, J.-L. Brédas, S. R. Marder, A. Kahn, B. Kippelen, *Science* **2012**, *336*, 327.

[2] K. Min, W. Choi, C. Kim, M. Choi, *Nat Commun* **2018**, *9*, 726.

[3] D. R. Baer, K. Artyushkova, H. Cohen, C. D. Easton, M. Engelhard, T. R. Gengenbach, G. Greczynski, P. Mack, D. J. Morgan, A. Roberts, *Journal of Vacuum Science & Technology A* **2020**, *38*, 031204.

[4] G. Greczynski, L. Hultman, *Progress in Materials Science* **2020**, *107*, 100591.

[5] Y. Xue, X. Li, H. Li, W. Zhang, *Nat Commun* **2014**, *5*, 4348.

[6] C. Passiu, A. Rossi, M. Weinert, W. Tysoe, N. D. Spencer, *Applied Surface Science* **2020**, *507*, 145084.

[7] P. H. Citrin, G. K. Wertheim, Y. Baer, *Phys. Rev. Lett.* **1978**, *41*, 1425.

[8] J.-P. Sylvestre, S. Poulin, A. V. Kabashin, E. Sacher, M. Meunier, J. H. T. Luong, *J. Phys. Chem. B* **2004**, *108*, 16864.

[9] P. Jiang, S. Porsgaard, F. Borondics, M. Köber, A. Caballero, H. Bluhm, F. Besenbacher, M. Salmeron, *J. Am. Chem. Soc.* **2010**, *132*, 2858.

[10] Z. Wang, X. Yang, Z. Yang, W. Guo, L. Lin, N. Li, E. Jiang, J. Zhang, B. Yan, J. Ye, *Frontiers in Materials* **2019**, *6*.

[11] J. Tang, H. Guo, M. Zhao, J. Yang, D. Tsoukalas, B. Zhang, J. Liu, C. Xue, W. Zhang, *Sci Rep* **2015**, *5*, 16527.

[12] B. S. Kim, H. Kwon, H. J. Kwon, J. B. Pyo, J. Oh, S. Y. Hong, J. H. Park, K. Char, J. S. Ha, J. G. Son, S.-S. Lee, *Advanced Functional Materials* **2020**, *30*, 1910214.

[13] E.-H. Ko, H.-J. Kim, S.-M. Lee, T.-W. Kim, H.-K. Kim, *Sci Rep* **2017**, *7*, 46739.

[14] D. Jung, C. Lim, H. J. Shim, Y. Kim, C. Park, J. Jung, S. I. Han, S.-H. Sunwoo, K. W. Cho, G. D. Cha, D. C. Kim, J. H. Koo, J. H. Kim, T. Hyeon, D.-H. Kim, *Science* **2021**, *373*, 1022.

[15] S. Lee, D. Sasaki, D. Kim, M. Mori, T. Yokota, H. Lee, S. Park, K. Fukuda, M. Sekino, K. Matsuura, T. Shimizu, T. Someya, *Nature Nanotech* **2019**, *14*, 156.

[16] J. Lee, S. Varagnolo, M. Walker, R. A. Hatton, *Advanced Functional Materials* **2020**, *30*, 2005959.

[17] C. Zhang, A. Khan, J. Cai, C. Liang, Y. Liu, J. Deng, S. Huang, G. Li, W.-D. Li, *ACS Appl. Mater. Interfaces* **2018**, *10*, 21009.

[18] J. H. Cho, D. J. Kang, N.-S. Jang, K.-H. Kim, P. Won, S. H. Ko, J.-M. Kim, *ACS Appl. Mater. Interfaces* **2017**, *9*, 40905.

[19] X. Chen, J. Chen, L. Huang, S. Nie, W. Xu, Y. Yin, S. Zhang, F. Pei, K. Yu, W. Su, Y. Wang, W. Yuan, Y. Li, Z. Cui, *Advanced Materials Technologies* **2023**, *8*, 2201406.

[20] S. Lee, G. Kang, J. Kang, H. Ko, *ACS Appl. Mater. Interfaces* **2023**, *15*, 53062.

[21] K. C. Topka, B. Diallo, M. Puyo, P. Papavasileiou, C. Lebesgue, C. Genevois, Y. Tison, C. Charvillat, D. Samelor, R. Laloo, D. Sadowski, F. Senocq, T. Sauvage, H. Vergnes, M.-J. Menu, B. Caussat, V. Turq, N. Pellerin, C. Vahlas, *ACS Appl. Electron. Mater.* **2022**, *4*, 1741.

[22] N. Damianakis, G. R. C. Mouli, P. Bauer, *Applied Energy* **2025**, *380*, 125000.

[23] X. Li, P. Li, Z. Wu, D. Luo, H.-Y. Yu, Z.-H. Lu, *Materials Reports: Energy* **2021**, *1*, 100001.

[24] N. T. Ho, S. Lee, B. S. Joo, J. Kim, J. Kang, J. H. Kim, G. Kang, H. Ko, *Laser & Photonics Reviews* **2024**, *18*, 2301178.
